# A molecular dynamics simulation study on the propensity of Asn-Gly-containing heptapeptides towards *β*-turn structures: Comparison with *ab initio* quantum mechanical calculations

**DOI:** 10.1371/journal.pone.0243429

**Published:** 2020-12-03

**Authors:** Dimitrios A. Mitsikas, Nicholas M. Glykos

**Affiliations:** Department of Molecular Biology and Genetics, Democritus University of Thrace, University campus, Alexandroupolis, Greece; University of Helsinki, FINLAND

## Abstract

Both molecular mechanical and quantum mechanical calculations play an important role in describing the behavior and structure of molecules. In this work, we compare for the same peptide systems the results obtained from folding molecular dynamics simulations with previously reported results from quantum mechanical calculations. More specifically, three molecular dynamics simulations of 5 μs each in explicit water solvent were carried out for three Asn-Gly-containing heptapeptides, in order to study their folding and dynamics. Previous data, based on quantum mechanical calculations within the DFT framework have shown that these peptides adopt *β*-turn structures in aqueous solution, with type I’ *β*-turn being the most preferred motif. The results from our analyses indicate that at least for the given systems, force field and simulation protocol, the two methods diverge in their predictions. The possibility of a force field-dependent deficiency is examined as a possible source of the observed discrepancy.

## Introduction

*β*-Turns are structural motifs defined by four consecutive residues (*i* to *i*+3) with the distance between the C^α^(*i*) and C^α^(*i*+3) atoms being less than 7 Å and where the central two residues are not helical (although a *β*-turn may overlap the end of an *α*-helix by up to three residues) [1–5]. They are typically classified into distinct categories based on the distribution of *φ* and *ψ* torsion angles of residues *i*+1 and *i*+2 [1–3]. Though many classification schemes have been proposed so far for *β*-turns [6], the almost universally accepted is the one proposed by Thornton [1] which defines nine distinct types of *β*-turns: I, I’, II, II’, VIa1, VIa2, V1b, VIII and IV. Type I and II *β*-turns are the most common types of *β*-turns found in proteins, with the ideal torsion angles (*φ*_*i*+1_, *ψ*_*i*+1_, *φ*_*i*+2_, *ψ*_i+2_) in each of these categories being (-60°, -30°, -90°, 0°) and (-60°, 120°, 80°, 0°), respectively, whereas, the corresponding values for their “mirror-image” type I’ and II’ *β*-turns are (60°, 30°, 90°, 0°) and (60°, -120°, -80°,0°) [1].

Although short peptides that appear in *β*-turns can be considered structurally as no well-behaved model systems, as they occasionally adopt random conformations, it is both the nature and position of specific residues that favors the propensity towards *β*-turn formation usually by stabilizing the formation of *β-*hairpin motifs [7–12]. Analyses of X-ray protein structures have provided valuable information about the positional preferences of the 20 amino acids in different types of *β*-turns [1–5, 7, 10–12]. Of particular interest are type I’ *β*-turns which are common among observed protein structures [1–3, 7]. The *φ*,*ψ* angles of this turn type are typically constrained in a small region of the Ramachandran plot for L-amino acids [1, 13]. Studies on the positional preferences of amino acid residues in positions *i*+1 and *i*+2 of type I’ *β*-turns indicate that, among others, Asn is generally favored in position *i*+1 and Gly in position *i*+2 [1–3]. Analysis of turns has shown that this major preference is not characterized by any distinctive bonding pattern [1], whereas results from NMR studies revealed that the Asn-Gly segment promotes the formation of type I’ *β*-turn and *β*-hairpin conformations, though not as effectively as the D-Pro-Xaa loop-forming segment [14–23]. Interestingly, thermodynamic analysis has suggested that the entropic advantage of the strong *β*-hairpin promoter sequence D-Pro-Gly is balanced to some extent by an enthalpic advantage of the Asn-Gly sequence, as the rigid nature of the D-Pro-Gly segment may prevent energetically favored contacts between side-chains [23]. Moreover, results from molecular dynamics analysis revealed no Asn side-chain interactions, but a pocket of “high-density” water involving the Asn side-chain and the peptide backbone, possibly playing an important role in modulating turn propensity [19]. However, although the Asn-Gly segment is found frequently in type I’ *β*-turns, no extensive computational investigation has been made on the folding of short peptides containing this segment. A recent study by Kang and Yoo [24], who examined the propensities of three Asn-Gly containing heptapeptides in aqueous solution to form *β*-turn structures using *ab initio* quantum mechanical (QM) calculations within the DFT framework, indicated that Asn-Gly type I’ *β*-turn was indeed the preferred structural motif for all the three heptapeptides. The optimized torsion angles for every *β*-turn type in each heptapeptide as obtained from their work [24] at the SMD M06-2X/6-31G(d) level of theory in water, are listed in Table 1. The resulting values are very similar to the ideal values proposed by Thornton’s work [1] without large discrepancies, apart from the *ψ*Gly value that despite residing within the ±30° deviation limit, has a slightly increased flexibility compared to the rest of the angles.

**Table 1:** Torsion angles (in degrees) of *β*-turn residues as obtained from the DFT-calculated structures.

Peptide	Turn type	*φ*_*i*_	*ψ*_*i*_	*φ*_i+1_	*ψ*_i+1_	*φ*_i+2_	*ψ*_i+2_	*φ*_i+3_	*ψ*_i+3_
**hp**_**NG**_**-1**	I	-64.5	165.4	-54.5	-37.0	-86.6	-12.3	-170.0	148.9
	I’	-86.0	92.2	53.3	41.5	98.7	-16.5	-77.8	92.5
	II	-62.1	163.9	-50.5	134.9	77.3	-6.9	-164.7	129.5
	II’	-84.3	96.0	51.9	-133.5	-71.3	-9.8	-80.5	90.2
**hp**_**NG**_**-2**	I	-106.0	153.7	-54.5	-35.1	-74.9	-26.2	-175.5	150.4
	I’	-132.4	119.6	57.8	36.1	91.2	-23.5	-80.0	109.3
	II	-108.6	151.0	-47.8	130.4	89.9	-18.1	-166.6	128.0
	II’	-130.1	132.5	57.1	-134.9	-87.8	-7.4	-84.1	108.9
**hp**_**NG**_**-3**	I	-130.0	168.9	-55.1	-28.3	-112.2	25.8	-158.4	145.3
	I’	-145.3	121.2	58.3	38.7	78.1	-4.8	-106.4	-169.9
	II	-129.7	167.6	-47.3	136.6	63.9	23.0	-160.0	130.6
	II’	-78.9	85.1	54.2	-146.9	-65.9	-24.8	-56.1	150.2

Folding molecular dynamics (MD) simulations using empirical force fields have matured to the point of becoming a powerful and useful analytical tool for studying the structure and dynamics of a wide variety of peptide systems. Our perspective on the current literature is that among all extensively tested and validated force fields [25], the AMBER99SB family of force fields and particularly the 99SB*-ILDN and—to a lesser extent—the 99SB-ILDN variants [26–29] show an outstanding performance against a wide range of systems, from very stable folders [26–31] to marginally stable peptides [30–37], and for every structural motif, from mainly helical [27–29, 31–33] to almost exclusively *β*-hairpin-like [29, 31, 37–39]. In this communication, we attempt to examine the accuracy of MD simulations and their ability to reproduce the results derived from the aforementioned DFT calculations (of ref 24), as well as experimental data (i.e., Thornton’s criteria) [1]. So far, data obtained from classical empirical MD force field analyses regarding the Asn-Gly segment are limited. The most notable finding that gave us cause for reflection on the folding of the Asn-Gly sequence was the inability of the AMBER99SB*-ILDN force field to fold and stabilize the Trpzip-2—which adopts an Asn-Gly *β*-turn motif [40]—possibly due to problems describing local conformational preferences [41, 42]. Therefore, using the same three Asn-Gly heptapeptides as Kang and Yoo, we studied their propensities to adopt *β*-turn conformations in water, and examined to what extent the AMBER99SB*-ILDN force field, combined with the TIP3P [43] water model, can provide accurate results regarding protein folding and dynamics, considering their hitherto successful application on a plethora of short and dynamically labile peptides. In the paragraphs that follow we discuss the design, and statistical significance of the calculations performed, and the results obtained by them, emphasizing on their comparison with the available DFT data, in connection as well with various experimental analyses. We conclude by discussing possible interpretations of our findings, concerning force fields convergence and development, and implementation of such theoretical computational approaches.

## Methods

### System preparation and simulation protocols

In order to study the propensities of the Asn-Gly segment to form *β*-turn structures we conducted three MD simulations for the same three capped heptapeptides that Kang and Yoo used in their work: Ac-Ala-Ala-**Asn-Gly**-Ala-Ala-NHMe (**hp**_**NG**_**-1**), Ac-Leu-Val-**Asn-Gly**-Gln-Tyr-NHMe (**hp**_**NG**_**-2**, from PDB entry 1EST) [44] and Ac-Phe-Val-**Asn-Gly**-Leu-Phe-NHMe (**hp**_**NG**_**-3**, derived from an octapeptide with the similar sequence Boc-Leu-Phe-Val-Aib-D-Ala-Leu-Phe-Val-OMe that forms a type I’ Aib-D-Ala *β*-turn) [45]. The system preparation procedure and simulation protocol have been previously described [30–38] and in summary were performed as follows. Starting from the fully extended states, addition of missing hydrogen atoms and solvation-ionization were performed with the LEAP program from the AMBER tools distribution. All three simulations were performed using periodic boundary conditions and a cubic unit cell sufficiently large to guarantee a minimum separation between the PBC-related images of the peptides of at least 16 Å. We followed the dynamics of the peptides’ folding simulations using the NAMD program [46, 47] for a grand total of 15 μs (5 μs for each peptide), using the TIP3P water model [43] and the AMBER99SB*-ILDN force field [28, 29], which has repeatedly been shown to correctly fold various peptides. For all the simulations, adaptive tempering [48] was applied as implemented in the NAMD program. Adaptive tempering is formally equivalent to a single-copy replica exchange folding simulation with a continuous temperature range. For our simulations this temperature range was 280 K to 480 K inclusive and was applied to the systems through the Langevin thermostat (see below).

The simulation protocol for all the peptides was the following: the system was first energy minimized for 1000 conjugate gradient steps followed by a heating-up phase to a final temperature of 320 K (with a Δ*Τ* step of 20 K) over a period of 32 ps. Subsequently, the system was equilibrated for 10 ps under NpT conditions without any restraints, until the volume equilibrated. This was followed by the production NpT runs with the temperature and pressure controlled using the Nosé-Hoover Langevin dynamics and Langevin piston barostat control method as implemented by the NAMD program, with adaptive tempering applied through the Langevin thermostat, while the pressure was maintained at 1 atm. The application of the adaptive tempering in combination with the choice of the NpT ensemble guarantees the absence of significant variations of the system’s mean density (and thus biologically relevant properties). The Langevin damping coefficient was set to 1 ps^-1^ and the piston oscillation period set to 200 fs with a decay time of 100 fs. The production run was performed with the impulse Verlet-I multiple time-step integration algorithm [49] as implemented by NAMD. The inner time-step was 2.5 fs, with short-range nonbonded interactions being calculated every one time-step, and long-range electrostatic interactions every two time-steps using the particle mesh Ewald method [50] with a grid spacing of approximately 1 Å and a tolerance of 10^−6^. A cutoff for the van der Waals interactions was applied through a switching function, acting between 7 and 9 Å. The SHAKE algorithm [51] with a tolerance of 10^−8^ was used to restrain all bonds involving hydrogen atoms. Trajectories were obtained by saving the atomic coordinates of the whole system every 1 ps.

### Trajectory analysis

The programs CARMA [52] and GRCARMA [53] together with custom scripts were used for most of the analyses, including removal of overall rotations/translations, calculation of torsion angles, calculation of the RMSDs from a chosen reference structure, production of PDB files from the trajectories, dihedral space principal component analysis [54–56] and corresponding cluster analysis, etc. Structural analysis was performed using the PROMOTIF [57] and PROCHECK [58] programs. All molecular graphics work and figure preparation were performed using the PyMOL [59] and CARMA programs.

### Torsion angle comparison analysis

A significant part of this work focused on analyzing the extent to which the *φ*,*ψ* angles obtained from the DFT calculations [24] and the ideal ones defined by Thornton’s work [1] agree with the *φ*,*ψ* angles derived from the MD simulations. Because the angles from molecular dynamics are continuously variable, this analysis was performed as follows. In the first step we calculated the deviations (in degrees) between the DFT-derived *φ*,*ψ* torsion angles (from ref 24) and their corresponding torsion angles from each frame of the trajectory (or from each frame of a dPCA-derived cluster). In the second step, the distributions of these angle deviations were calculated and the resulting histograms plotted. In the final step, we treated these distributions as a sum of independent Gaussian distributions, and we obtained numerical values for the means (*μ*) and standard deviations (*σ*) of those peaks residing closest to the reference DFT angle values (the calculation of the (*μ*, *σ*) parameters was performed with the *nlsLM* function of the *R* package) [60]. These values were then used to convert the calculated deviations to probabilities, as will be discussed later.

## Results and discussion

### Extent of sampling

**Fig 1:**
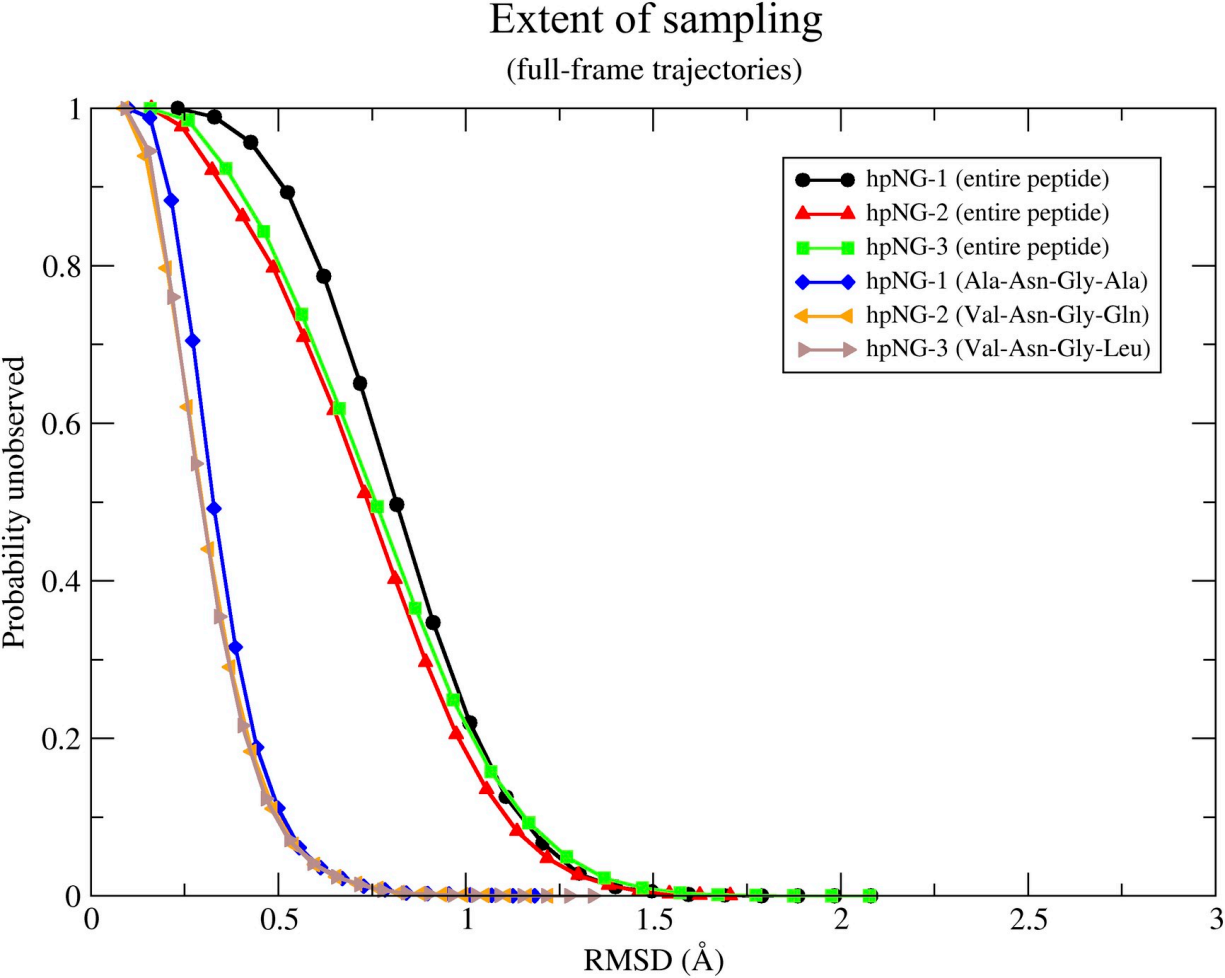
Extent of sampling and statistical significance. Results from the application of Good-Turing statistics to the three trajectories for both the full-length peptides and their four-residue central part. See text for details.

The heptapeptides—as will be discussed in the next section—demonstrate a highly dynamic behavior throughout the molecular dynamics simulations. Their structural flexibility/disorder leads to a rugged folding landscape and, consequently, to the absence of a well-defined gradient towards a would-be ‘native’ structure. The implication of this finding is that the whole configurational space available to the peptides is accessible during the simulations. This immediately raises the issue of convergence and statistical significance of our results. To tackle this problem, we apply a recently proposed probabilistic method [61] for estimating the convergence of molecular dynamics trajectories. The method is based on the application of Good-Turing statistics to estimate the amount of structural variability that has *not* been observed in a given trajectory. These probabilistic estimates are presented in the form of graphs that depict the probability of observing a new/different structure, as a function of the minimal RMSD—of this (thus far unobserved) structure—from all those structures already observed. The results from the application of Good-Turing statistics are shown in Fig 1.

To clarify these graphs, the high probability of unobserved species for low RMSD values indicates that if we were to continue the simulation (even for just one additional time-step) it would be highly probable to observe structures, which although similar to some of the already observed ones, they would still be slightly different from them. As the RMSD from the already observed structures increases, the corresponding probability decreases. It is therefore the exact form of this graph and the inclination of each curve towards low probability values that inform us to what extent the trajectories have been sampled or, in other words, how significant is the structural variability that has been missed due to limited sampling. Focusing on the application of Good-Turing statistics in our trajectories, the results have been obtained from multiple calculations and are organised in two groups. Fig 1 illustrates the direct application of the method to the MD trajectories considering only the backbone atoms for either the entire heptapeptides (upper three curves) or their four-residue central part (lower three curves). For the entire peptides, the statistical analysis implies that if we continued the simulations, we would expect that on average 1/10 of the new (previously unobserved) structures would differ by an RMSD of at least ~1.3 Å. Clearly, the effect of limiting the residue selection to the amino acids forming the *β*-turn is rather dramatic: the curves fall quite fast to negligible *p*_unobserved_ values for RMSDs of the order of 1 Å. The apparent difference between these two cases may be attributed to the fact that the four-residue central parts are structurally more stable, possibly promoting the formation of secondary-structure-like patterns, whereas the peptides' termini are highly mobile and disordered.

To conclude, the results drawn from the analysis of our 5 μs trajectories indicate that the length of the simulations is probably sufficient and guarantees a reasonable sampling of the peptides’ configurational space for the given force field. A similar type of analysis has also been made but using only structures whose corresponding adaptive tempering temperature was ≤ 360 K, thus corresponding to more stable (from the simulation’s point of view) conformers. As shown in S1 Fig, the differences are minor compared to the first analysis and setting a temperature cutoff for examining only more stable peptide conformers does not result in our case in a better sampling of the configurational space.

### The peptides are very flexible, but with a tendency to form *β*-turns

**Fig 2:**
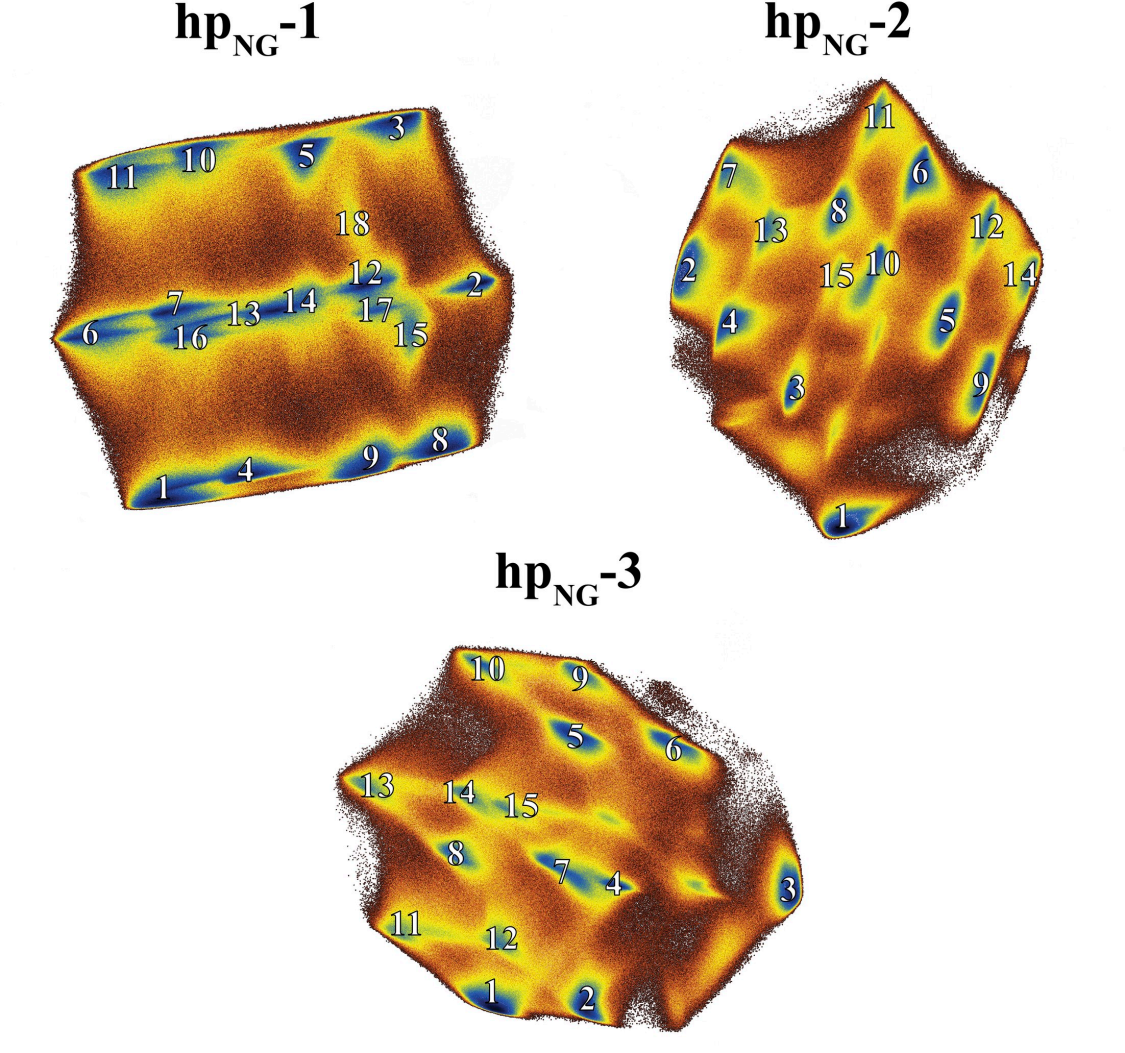
Dihedral angle principal component analysis. Diagrams illustrating the free energy landscape of each heptapeptide along the top two principal components, as obtained by the dihedral angle Principal Component Analysis (blue peaks correspond to high density regions). Shown with numbers are the different clusters derived by the dPCA analysis.

**Fig 3:**
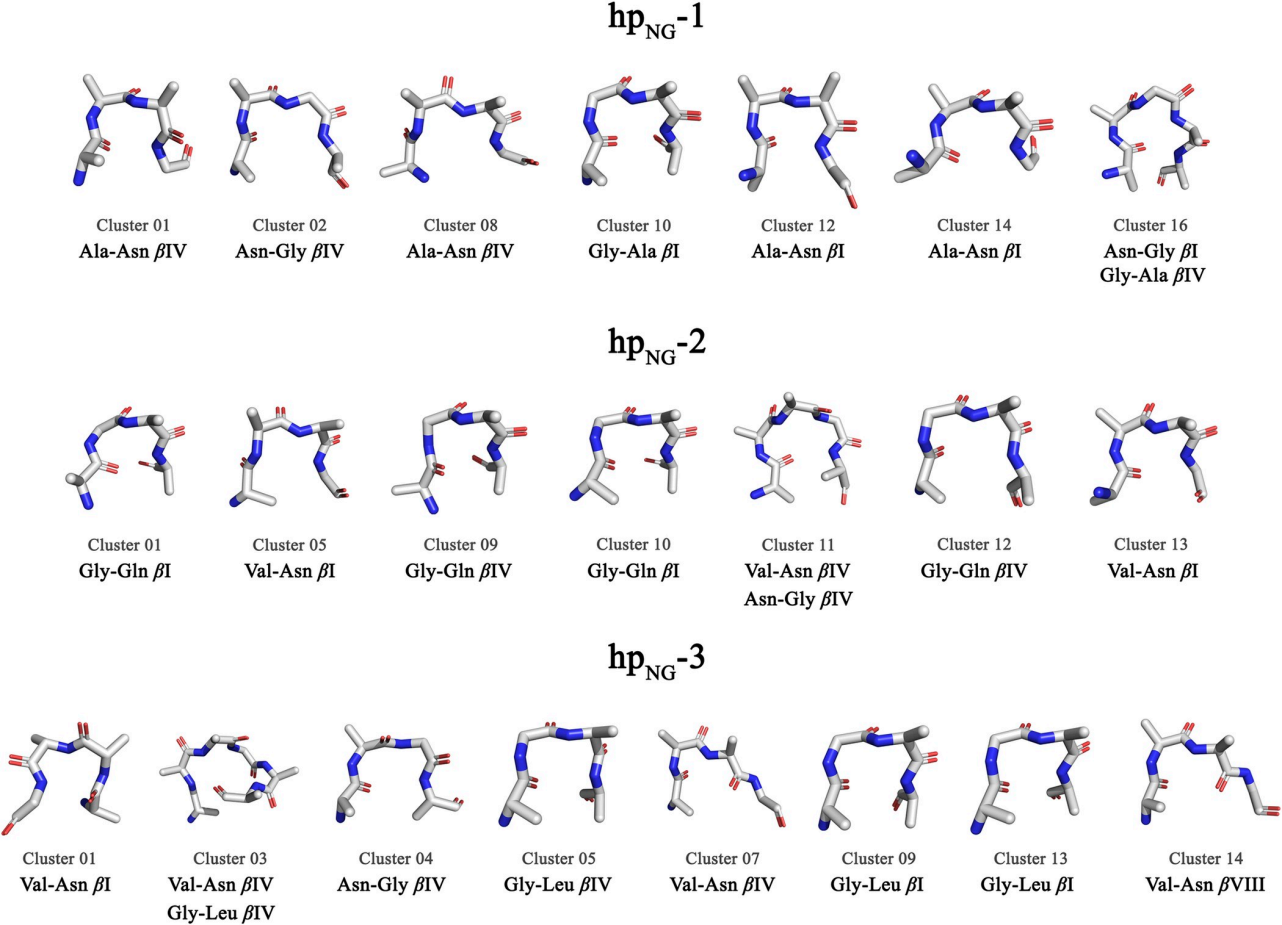
dPCA-derived *β*-turn representative structures. Clusters’ backbone plus C^β^ representative structures that correspond to *β*-turn motifs. The structural analysis was performed using the PROMOTIF program and structures were generated using the PyMOL program. The color coding used here is representative of the atoms’ type (blue color corresponds to N atoms, red color corresponds to O atoms and light grey color corresponds to C atoms). Shown are only representative *β*-turn structures that correspond to clusters of population > 1%.

To place our observations on a structurally firm ground, we calculated the free energy landscapes of the heptapeptides using the dihedral angle Principal Component Analysis (dPCA) method to identify the prominent conformations. Various studies have already indicated that dPCA is a high resolution, powerful and more appropriate method for studying such highly flexible systems [54–56]. We restricted our analysis to the backbone dihedral angles of the four central residues only, since the rest of the peptides were found to be kinetically frustrated and uncorrelated to the rest of the system. The results from the dPCA analysis are shown in Fig 2 in the form of a set of diagrams illustrating the log density projections of the corresponding trajectories along the top two principal components (i.e., the free energy landscapes). High density peaks (dark blue regions) can be associated with distinct peptide conformers. Representative structures of these clusters were extracted after calculating an average structure of each cluster and then selecting the frame of the trajectory with the lowest RMS deviation from the corresponding average structure. Representative structures corresponding to *β*-turn motifs for each heptapeptide can be seen in Fig 3. Νote that the actual cluster analysis was performed in the three-dimensional principal component space, and not in two dimensions as shown here for clarity.

As it can be seen in Fig 2, the free energy landscapes are quite rugged with several free energy minima that correspond to numerous distinct conformations, rather than a few stable and persistent structures. For **hp**_**NG**_**-1**, 34.7% of the total frames were assigned by the program CARMA to clusters, while for **hp**_**NG**_**-2** and **hp**_**NG**_**-3** the corresponding percentages were 36.7% and 31.8%, respectively. The most highly populated cluster recorded for each heptapeptide occupied only 11.9%, 20.1% and 20.7% of the clustered frames (for the **hp**_**NG**_**-1**, **hp**_**NG**_**-2** and **hp**_**NG**_**-3** respectively), clearly indicating that the peptides demonstrate a very dynamic behavior. S1 Table offers a detailed view regarding the population (among clustered frames) of each cluster and the occupancy of *β*-turn motifs in each cluster. There is a structural variability concerning the preference among turn types and the positional preference of each amino acid residue in positions *i*+1 and *i*+2. Asn-Gly turns, not only failed to be the dominant structural motif, but the analysis also revealed the presence of a plethora of *β*-turn motifs as well. Examination of clusters’ representative structures reveals that structures can vary among different *β*-turn and unfolded conformations. Fig 3 shows a collection of those clusters’ representative structures that form a *β*-turn, clearly demonstrating the large diversity of the turn types that have been sampled. Many representative structures corresponding to significantly populated clusters adopt a *β*-turn conformation, but the presence of unfolded random coil representatives is nonetheless considerable. Asn-Gly is not favored to be the turn segment among the representatives, as there is a variety in the positional preference of each residue for this site.

To have a more comprehensive understanding of the preferences among the different *β*-turn motifs, Table 2 shows the populations of *β*-turns sampled in each trajectory taking into account both the different combinations of *i*+1 and *i*+2 positional preferences and the different types of *β*-turns. Of the turns located, *β*IV turns seem to be the most prominent turn motif in each trajectory, but this can be attributed to the fact that this miscellaneous type does not follow any of the stringent criteria being used for turns’ classification scheme [1, 6]. *β*VIII turns’ sampling on the other hand is limited, resulting only in a negligible amount of observations. As for the most common classical turn motifs *β*I, *β*I’, *β*II, *β*II’, we must also focus on the various analyses of turns derived from experimental data, apart from the DFT calculations, so that we examine the possibility that our simulations have accurately described the peptides’ folding behavior. X_-1_-Asn and Gly-X_+1_ *β*I turns appear to be the most frequently sampled turn motifs in each trajectory, in agreement with the overall positional potentials which show a distinctive preference for Asn in positions *i* and *i*+2 [1–5, 7, 10]. Specifically for Asn, the side-chain can act as a hydrogen bond acceptor at position *i* forming a hydrogen bond with the main-chain nitrogen of residue *i*+2, stabilizing consequently type I *β*-turns [1]. In this case, residues *i* to *i*+3 form another turn-like structure known as the “Asx-turn”, which is commonly observed in type I *β*-turns [1, 62]. Additionally, Asn residues are likely to adopt the correct conformation (*φ* = -90°, *ψ* = 0°) for position *i*+2 in *β*I turns [1–3]. These findings, along with the flexible nature of Gly and its strong preference for the *α*_L_ conformation in position *i*+3 of type I turns [1–3], might be able to explain the high populations of X_-1_-Asn and Gly-X_+1_ *β*I turns in our trajectories. Regarding type II’ turns, although poorly sampled, especially in the case where Asn-Gly occupies the turn position, Gly-X_+1_ has a relatively increased preference as a turn segment for this turn type, in agreement with the crystallographic data where Gly shows a major preference for position *i*+1 in *β*II’ turns due to *φ*,*ψ* restrictions around +60°, -120° respectively [1]. Additional findings from NMR studies imply the presence of *β*II’ Gly-X turns in model peptides containing the Asn-Gly segment [20]. Likewise, the preceding Asn residue might be involved in an Asx-turn motif, which is most frequently found in *β*II’ turns (followed by *β*I turns) [62], justifying as well the notable Gly-X_+1_ populations. Finally, the Asn-Gly segment is slightly to moderately favored as a turn segment depending on the heptapeptide, showing a rather increased preference towards *β*II and *β*I’ turns (for **hp**_**NG**_**-1** Asn-Gly *β*I turns are also considerably preferred). Although Gly residue very often occupies the *i*+2 position in both of the aforementioned turn types (as it most readily adopts the *α*_L_ and *γ*_L_ conformations) [1, 7], the Asn residue has only been vaguely observed in position *i*+1 of *β*II turns in protein structures [1–5]. In *β*I’ turns contrarily, the Asn residue occurs very frequently in this position, as several studies have already indicated [1–5, 16–20, 23], possibly due to *φ*,*ψ* constraints around the *γ*_L_ region of the Ramachandran plot [1, 7]. Having made these observations, however, the question naturally arises as to their significance. For the specific case considered here, our comparisons might be inconclusive: as solution-phase experimental data (e.g. from NMR) are limited regarding these peptide systems, the usage of solid-state results (i.e., X-ray structures from database) to validate if a force field adequately samples torsion angles and conformations in solution phase over a large temperature range, might not be entirely proper. Yet, the take-home message of this analysis is clear: no dominant presence of Asn-Gly *β*I’ turns is apparent in our results, contrary to the consistent intrinsic preference of this dipeptide for the turn segment in *β*I’ turns as shown by numerous above-mentioned studies.

**Table 2:** Populations (%) of the various *β*-turn types sampled in the MD trajectories^a^.

		hp_NG_-1			hp_NG_-2			hp_NG_-3	
*β*-Turn type^b^	Ala-Asn	Asn-Gly	Gly-Ala	Val-Asn	Asn-Gly	Gly-Gln	Val-Asn	Asn-Gly	Gly-Leu
**I**	9.71	3.20	8.16	10.73	3.88	9.04	12.46	4.60	8.86
**I’**	0.41	2.34	0.38	0.28	6.22	0.19	0.24	5.64	0.17
**II**	0.62	5.36	0.81	0.75	5.55	0.31	0.89	6.19	0.28
**II’**	0.17	0.04	7.35	0.06	0.03	6.35	0.06	0.03	3.70
**VIII**	1.51	0.13	0.40	1.07	0.10	0.43	0.96	0.12	0.36
**IV**	12.47	8.12	13.12	16.24	9.19	17.18	15.17	8.95	17.22
**Total**	24.89	19.19	30.22	29.12	24.97	33.49	29.78	25.54	30.58

^a^ Shown are the % trajectory populations of the different *β*-turns containing Asn/Gly as one of their central residues. The assignment of *β*-turns was performed using the PROMOTIF program.

^b^ Types VIa1, VIa2 and VIb are excluded from our analysis as they require a Pro residue in position *i*+2.

Our findings, on the other side, do not straightforwardly agree with the results from the QM calculations performed by Kang and Yoo, at least in respect to the absolute structural propensities of the Asn-Gly sequence. As stated in their work [24], the most favored conformation for all three heptapeptides according to their calculations is the Asn-Gly *β*I’ turn motif (for **hp**_**NG**_**-1** *β*II’ and *β*I’ turn motifs were almost equally preferred). In contrast, the present MD analysis shows that Asn-Gly turns are overall (Table 2) the least sampled turn motif with their populations reaching approximately 20–25% of the total frames in each trajectory, contrary to those of X_-1_-Asn and Gly-X_+1_ turns that correspond to approximately 30% of total frames. In general, the results from the molecular dynamics simulations indicate that although the presence of *β*-turn motifs is a very significant feature of the peptides’ structural behavior, the trajectories are also characterized by a significant structural diversity, rather than a dominant presence of only type I’ *β*-turns or an exclusive preference for the Asn-Gly sequence as the turn segment.

Although the general pre-existing conformational preferences of the amino acid residues are important for the turn formation, side-chain to side-chain or side-chain to backbone interactions also play a key role in the folding and stabilization of turns and by extent hairpin motifs, especially in the context of a three-dimensional protein structure [8–12, 19–23]. However, the highly conserved Asn-Gly segment in type I’ *β*-turns does not show any side-chain interactions [7, 19, 20, 24]. The Asn side-chain in position *i*+1—especially the χ_2_ angle—is considered to be highly variable precluding any electrostatic interactions, especially with the residue’s amide group, hence reducing the bias for the *ψ* angle of the preceding residue to be compatible with the *β*-hairpin motif and by extend “primed” *β*-turn conformations [7–9, 63, 64]. This case may also explain the notable Asn-Gly *β*I populations that have been sampled. Additionally, the importance of hydrophobic interactions in stabilizing *β*-hairpin structures has also been examined in earlier studies [19–23, 64–68]. Though their role in affecting the formation of turn sequences remains controversial [7, 18, 64, 65], when such short and flexible peptides are studied, they might have a meaningful impact—along with hydrogen bonding interactions—on the thermodynamic stability of the system. The absence of long flanking sequences and, consequently, of enough hydrophobic interactions in our peptide systems might possibly further decrease the stability of the folded conformations, giving rise to a variety of *β*-turn motifs. Similarly, despite the presence of amino acid residues suitable for the incorporation in the strand segments of *β*-hairpins (such as Val, Tyr, Phe and to a lesser extent Leu), these residues do not occupy proper pair-facing positions that strongly influence stabilization interactions [11]. Finally, the high intrinsic propensity of the Asn-Gly segment towards *β*I’ turns is not unequivocally established, as previous NMR and MD studies implied either the concomitant presence of other turn motifs [20] or the inability to properly fold the peptides when the Asn-Gly segment was incorporated [66].

Having said the above and seeing our results in a positive light, given that MD simulations are superior in sampling a large conformational phase space, they might adequately interpret at first sight the behavior of such short and flexible peptides isolated in water solvent. Despite arguing in favor of our MD results though, our aim at this point is not to dispute the validity of the QM calculations, but to note that generating single structures on the *ab initio* level might overshadow the structural distribution of these systems.

### Torsion angles derived from MD generally agree with the DFT-obtained and the ideal *β*-turn *φ*,*ψ* values, but significant discrepancies are present

**Fig 4:**
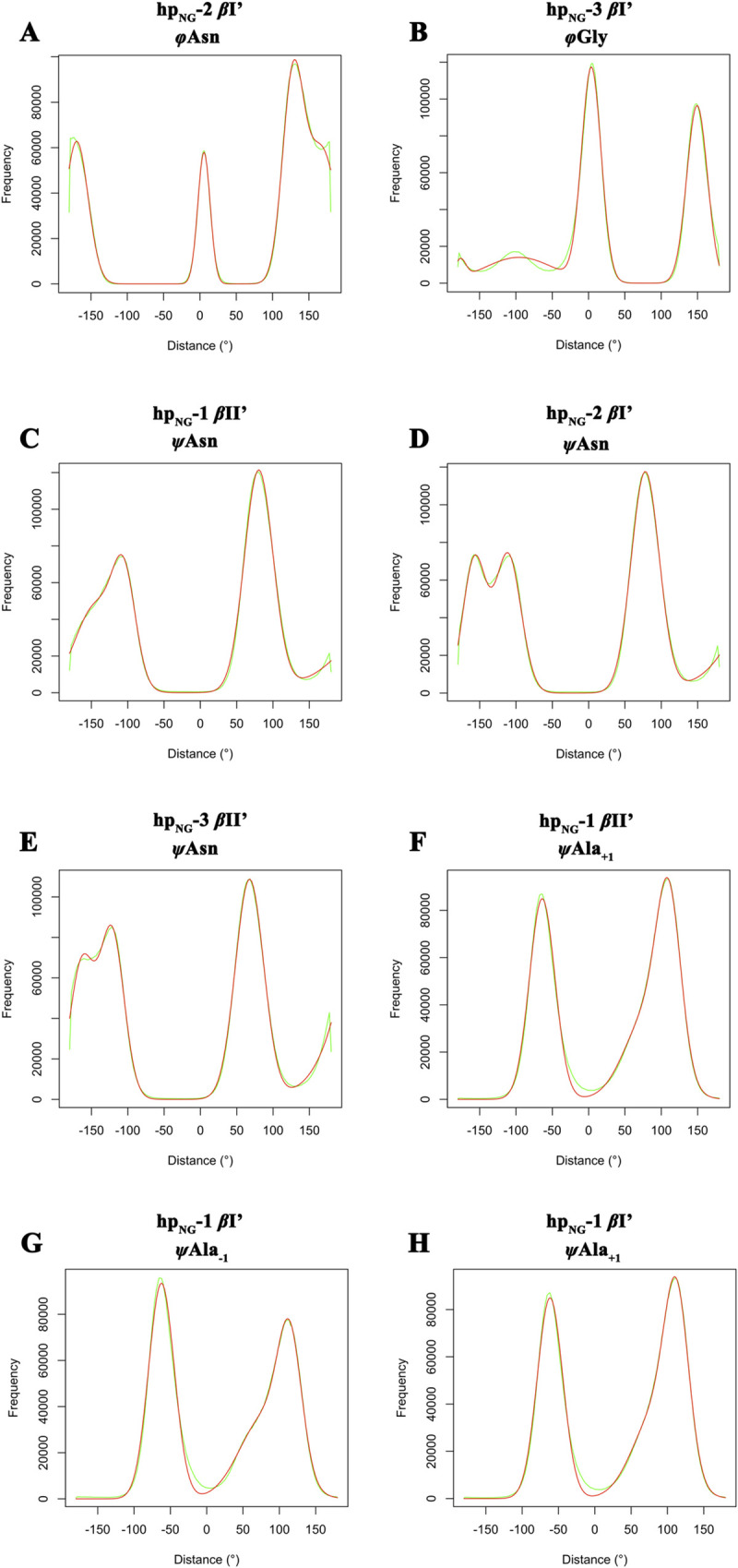
Distribution of deviations (in degrees) between MD trajectory-derived and DFT-calculated *φ*,*ψ* angles. Histograms showing the distribution of deviations (in degrees) between a reference DFT-obtained *φ*,*ψ* value and the corresponding *φ*,*ψ* values obtained from the MD simulations for selected residues. While there are torsion angles of specific residues that fluctuate around the DFT values (*d* = 0°) during the simulation [Gaussian distributions in the middle of panels (A)-(B)], some torsions present a significant divergence from the proposed values (C-H).

**Fig 5:**
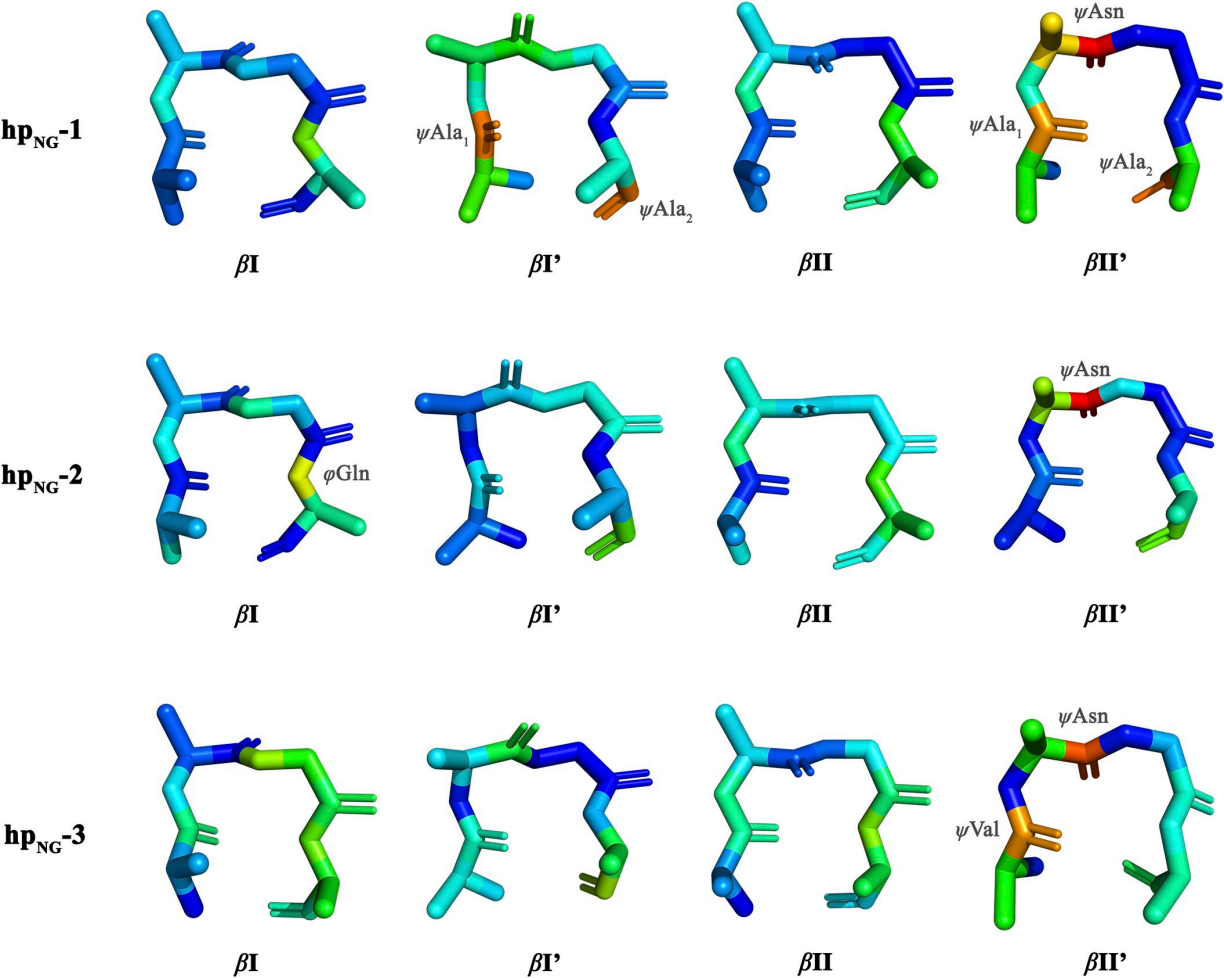
DFT-derived structures colored according to the magnitude of deviation from MD simulations. Shown are the DFT-obtained structures illustrating the torsion angle discrepancies in relation with the MD-derived angles. The color coding is indicative of the mean deviation of the MD *φ*,*ψ* values from the corresponding DFT-obtained ones, varying from blue (small deviations) to green (moderate deviations) and red (large deviations). Shown with a label, are the torsion angles presenting the greatest deviation throughout the simulations.

In the previous section we examined the general structural characteristics of the simulations in terms of preferred peptide conformers and turn types. In this section we make a more detailed comparison with the DFT results [24] through a direct numerical evaluation of the differences observed for individual torsion angles. The procedure is based on calculating for each frame (and for each of the trajectories) the torsion angles of the central four-residue part of the heptapeptides, followed by the calculation of the deviation (in degrees) between the MD-derived angles and the DFT-derived ones (from ref 24). Fig 4 shows the results in the form of histograms illustrating the deviation of each torsion angle from the corresponding DFT one. Values near *d* = 0° indicate similarity with the DFT model (as the deviations between the values of the MD torsion angle and the corresponding DFT values are small), while values far from *d* = 0° indicate large deviations. To further quantify these observations, we treated the distributions of these deviations (green curves in Fig 4) as a sum of independent Gaussian distributions, and we located the Gaussian distributions closest to the DFT angle values (i.e., the *d* = 0°) and obtained numerical values for their means (*μ*) and standard deviations (*σ*) through a nonlinear regression fitting performed with the *nlsLM* function of the *R* package (red curves in Fig 4). Using these values we then calculated Z-scores and the corresponding probabilities for the observed deviations between the QM-derived peptide structures and the molecular dynamics-derived ones. The numerical results are shown in S2 Table. In this table, large Z-scores (and their associated low probabilities) signify a statistically important deviation between DFT calculations and MD. Overall the agreement between the two methods appears to be reasonable but with some notable exceptions. On one hand, almost half of the MD-derived values lie within one standard deviation from the DFT reference angle values, while a significant number of them lie within two standard deviations. This implies that the majority of the DFT *φ*,*ψ* angle values have also been sampled by the molecular dynamics simulations giving rise to local conformations that are similar to the QM-derived *β-*turn structures. On the other hand, there are angles exhibiting large deviations from the DFT values. As we can see in Fig 4, while there are torsions sampled throughout the trajectories similar to the DFT angles (Gaussian distributions in the middle of panels A-B of Fig 4), in some cases examined, the 99SB*-ILDN force field failed not only to reproduce, but even to sample otherwise allowed values (as denoted by the structural evaluation using the PROCHECK program in S2–S4 Figs). Such examples are shown in panels C-H of Fig 4. Table 3 shows an overview of all torsion angles for which their mean values, as derived from molecular dynamics, deviate by more than 30° from their corresponding DFT-calculated values. To aid visualization of these findings, we show in Fig 5 the DFT-derived structures but colored according to the magnitude of deviation between the DFT angles and the simulation derived ones. The color coding in Fig 5 is indicative of the MD-DFT deviations and ranges from blue (small deviations) via green (moderate deviations) to red (large deviations). Torsion angles that demonstrate the largest deviations throughout the simulations are shown labelled. Despite the fact that there are obvious, but nonrecurring, discrepancies in some angle values between the MD and the DFT conformations (mainly regarding flanking residues), the MD angle values converge to some extent with the DFT-derived ones. However, *ψ*Asn angles from the *β*II’ conformations are the only angles that demonstrate a persistent and consistent deviation (shown also in panels C-E of Fig 4), denoting the inability of the force field to successfully sample and fold *β*II’ Asn-Gly turns, as also presented above in Table 2 populations.

**Table 3:** MD torsion angles exceeding the ±30° deviation limit from the DFT-calculated angles, along with their corresponding deviations^a^ in terms of absolute value.

hp_NG_-1		hp_NG_-2		hp_NG_-3	
*angle*	*deviation*	*angle*	*deviation*	*angle*	*deviation*
*φ*Ala_2_ *β*Ι	|39.6°|	*φ*Gln *β*Ι	|45.9°|	*φ*Gly *β*Ι	|41.2°|
*ψ*Ala_1_ *β*Ι’	|62.3°|	*ψ*Gln *β*Ι’	|38.4°|	*φ*Leu *β*Ι	|38.6°|
*ψ*Asn *β*Ι’	|33.0°|	*φ*Gln *β*ΙΙ	|36.8°|	*ψ*Leu *β*Ι’	|42.2°|
*ψ*Ala_2_ *β*Ι’	|61.6°|	*ψ*Asn *β*ΙI’	|77.9°|	*φ*Leu *β*ΙΙ	|40.2°|
*φ*Ala_2_ *β*ΙΙ	|34.3°|	*ψ*Gln *β*ΙI’	|38.8°|	*ψ*Val *β*ΙI’	|58.4°|
*ψ*Ala_1_ *β*ΙΙ’	|58.5°|			*ψ*Asn *β*ΙI’	|67.6°|
*ψ*Asn *β*ΙΙ’	|80.3°|				
*ψ*Ala_2_ *β*ΙΙ’	|63.9°|				

^a^ Deviations equal to the mean value (*μ*) of the Gaussian distribution located closest to the corresponding DFT reference angle value (i.e., *d* = 0^o^).

Closer examination of Fig 4 and Table 3 shows that the distributions of these *β*ΙΙ’ *ψ*Asn angles from the simulations cluster around three distinct regions. The large peak [right-hand side peak in panels C-Ε of Fig 4] corresponds to an area (in the Ramachandran plot) centered around *ψ ≈* +145° while the two shorter peaks [left-hand side of panels C-Ε of Fig 4] correspond to areas centered on *ψ ≈* +15° and *ψ ≈* -25°. These areas are close to the proposed values of *ψ* = *+*120° for the position *i*+1 in *β*II turns, *ψ* = ±30° for the position *i*+1 in *β*I and *β*Ι’ turns and *ψ* = 0° for the *i*+2 positions of all *β*-turn types. These results not only agree, but also explain both the presence of significant populations of X_-1_-Asn turns and Asn-Gly *β*Ι, *β*Ι’ and *β*ΙΙ turns, as well as the almost complete absence of Asn-Gly *β*II’ turns from the trajectories.

To complete this set of comparisons, we also calculated the deviations (in degrees) between the mean Asn/Gly torsion angles from molecular dynamics and the corresponding *φ*,*ψ β*-turn values from Thornton’s [1] definitions. The results are shown in Table 4 and confirm and reinforce the conclusions drawn from the comparison with the DFT values: almost all of these torsion angles from the trajectories fluctuate around the ideal *φ*,*ψ* values (with their mean values residing within the deviation limit of ±30°). The outliers are again *ψ*Asn in *β*II’ **hp**_**NG**_**-1**, *ψ*Asn in *β*II’ **hp**_**NG**_**-2** and *ψ*Asn in *β*II’ **hp**_**NG**_**-3**, which show a very significant deviation from the ideal *β*-turn values, far exceeding the ±30° limit set by Thornton and coworkers.

**Table 4:** Absolute differences between the calculated Gaussian mean values (*μ*)^a^ and the ideal *β*-turn *φ*,*ψ* torsion angles, for Asn/Gly residues of each turn type.

Turn type	hp_NG_-1	hp_NG_-2	hp_NG_-3
***β*Ι**	*φ*Asn |14.9°|	*φ*Asn |13.1°|	*φ*Asn |12.1°|
	*ψ*Asn |2.1°|	*ψ*Asn |3.3°|	*ψ*Asn |0.4°|
	*φ*Gly |18.2°|	*φ*Gly |19.5°|	*φ*Gly |19.0°|
	*ψ*Gly |5.4°|	*ψ*Gly |1.9°|	*ψ*Gly |3.0°|
***β*Ι’**	*φ*Asn |28.3°|	*φ*Asn |7.8°|	*φ*Asn |7.2°|
	*ψ*Asn |21.5°|	*ψ*Asn |9.3°|	*ψ*Asn |19.9°|
	*φ*Gly |17.7°|	*φ*Gly |18.1°|	*φ*Gly |15.8°|
	*ψ*Gly |5.4°|	*ψ*Gly |1.9°|	*ψ*Gly |3.0°|
***β*ΙΙ**	*φ*Asn |14.9°|	*φ*Asn |13.1°|	*φ*Asn |12.1°|
	*ψ*Asn |26.0°|	*ψ*Asn |26.9°|	*ψ*Asn |25.6°|
	*φ*Gly |8.1°|	*φ*Gly |8.1°|	*φ*Gly |5.8°|
	*ψ*Gly |5.4°|	*ψ*Gly |1.9°|	*ψ*Gly |3.0°|
***β*ΙΙ’**	*φ*Asn |31.1°|	*φ*Asn |7.8°|	*φ*Asn |7.2°|
	*ψ*Asn |93.1°|	*ψ*Asn |92.8°|	*ψ*Asn |94.51°|
	*φ*Gly |8.2°|	*φ*Gly |9.5°|	*φ*Gly |8.9°|
	*ψ*Gly |5.4°|	*ψ*Gly |1.9°|	*ψ*Gly |3.0°|

^a^ The Gaussian mean values (*μ*) correspond to the mean values of these Gaussian distributions lying closer to *d* = 0^o^ (i.e., the reference DFT angle values).

Although, as mentioned above, the results from our MD simulations, under the scope of the application regime they have been designed for, manifest a substantial convergence with data concerning the general amino acid frequencies in turns, such conspicuous disparity with the experimental/DFT results regarding *ψ*Asn *β*II’ angles disputes the validity of our simulations. The fact that molecular dynamics utterly fail to sample energetically allowed—as indicated by QM calculations—angle values, along with the absence of a strong propensity towards the Asn-Gly *β*I’ conformation, might underlie a force field discrepancy. Indeed, previous studies on the Asn-Gly *β*-hairpin Trpzip-2 model peptide using the 99SB*-ILDN force field, showed the inability of this force field variant to sufficiently fold and stabilize the *β*-hairpin conformation [41, 42]. This possibility of a force field induced discrepancy will be more extensively discussed in a separate section later in the text.

### Torsion angle analysis of the dPCA-derived clusters confirms and reinforces the results from the entire trajectories

To compare our previous observations with the results obtained from the dPCA we calculated, as described above, the deviations of the *φ*,*ψ* angles in each cluster’s frame from the corresponding DFT-calculated value. S5–S7 Figs show—for the five most populated clusters of each heptapeptide—the distributions of these deviations for the torsion angles of the four-residue central part, together with the occupancy of the corresponding *β*-turns and a representative structure for each cluster. A closer look at the histograms reveals a significant divergence of the Asn/Gly *φ*,*ψ* peaks from the *d* = 0° value for several clusters, showing that Asn-Gly turns have not been adequately sampled in the major dPCA clusters. The cases where this torsion angle analysis indicates convergence with the DFT data can also be verified through a comparison with the results shown in S1 Table. Clusters 09 and 03 of **hp**_**NG**_**-1**, for example, have significant populations of Asn-Gly type II and I *β*-turns respectively, as the sharp Asn/Gly *φ*,*ψ* peaks lying around *d* = 0° indicate, but the Asn-Gly *β*I’ motif has not been sampled at all (S5 Fig). Clusters 01 and 02 of **hp**_**NG**_**-2** have notable populations of Asn-Gly *β*I’ and *β*I turns respectively (S6 Fig), whereas for **hp**_**NG**_**-3** clusters 01, 03 and 07 are considerably populated by Asn-Gly *β*I, *β*I’ and *β*ΙΙ turns respectively (S7 Fig). S8 Fig shows in the form of barcharts the occupancy of Asn-Gly turns for each trajectory and cluster, based on a PROMOTIF analysis of a sample of 500 randomly selected structures derived from each dPCA cluster. Bars are colored according to the type of different *β-*turn motifs, and indicate the percentage (out of these 500 structures) of Asn-Gly turns sampled in the clusters. The results are in good agreement with the previously observed populations of S1 Table and S5–S7 Figs. Asn-Gly turns are indeed preferred to some extent by our peptide systems, but they are not the major structural motifs observed. Some of the major clusters are not even occupied by Asn-Gly turns indicating that many Asn-Gly turn conformers may have been sampled throughout the trajectory but do not belong in any of the dPCA-derived clusters. Similarly, no populations of Asn-Gly *β*II’ turns have been identified. As for the Asn-Gly *β*Ι’ turn, although it is present, it is definitely not a predominant structural motif of the dPCA clusters. A question that arises at this point is this: Can the clusters be characterized by a single structural motif? In other words, can a highly populated motif be representative of a cluster’s structural ensemble? If we look in S5–S7 Figs, even though there are clusters with significant populations of a certain *β*-turn type, they cannot be unequivocally identified by a single motif. As can be seen in S5–S7 Figs, representative structures differ from the general structural propensities that are apparent in each cluster. Structural analysis of the representative conformations shown in Fig 3 compared with the results presented in S1 Table also indicate that representative structures may adopt conformations that differ significantly from the heavily populated structures identified in each cluster. In a sense, this variability is not unexpected: the analysis in S5–S7 Figs attempts to characterize the structural motifs observed in the whole of the heptapeptides but based on the clustering obtained from a dPCA analysis of only the four central residues. The reason for our choice to focus the analysis on the four central residues is, of course, that it simplifies the comparison with the QM data which is the principal target of this investigation.

To summarize, turn populations in clusters are indicative of what is mentioned above: the small fraction of stable, folded *β*-turns in each cluster gives rise mainly to coil or non-classical *β*IV representative structures, suggesting that clusters are barely characterized by native-like conformations. While the tendency towards *β*-turn formation is in general present, the structural diversity observed in our trajectories (and clusters) precludes us from identifying adequately large populations of specific turn motifs that would be indicative of a propensity towards specific *β*-turn structures, such as the type I’ Asn-Gly turns. That being said, along with the incapability of the force field to sample certain torsion angles—and therefore conformations—identified as energetically favoured by the DFT calculations [24], cast doubts on the accuracy of the applied force field.

### Possible deficiencies in both MD simulations and DFT calculations are examined

As previously mentioned, the inability to sample Asn-Gly *β*II’ turns that—although meagerly reported in previous statistical analyses of database [1–5, 7]—have been identified as energetically prominent conformations by the DFT calculations (in ref 24), may imply a force field discrepancy. Additionally, the lack of strong evidence indicating a heavy intrinsic propensity of the Asn-Gly segment to form *β*I’ turns, as previously has been confirmed by several experimental and statistical analyses [1–5, 16–20, 23], might also be attributed to such force field deficiencies. Previous data have already shown that the AMBER99SB*-ILDN force field failed to adequately fold and stabilize another Asn-Gly *β*-hairpin peptide, the Trpzip-2, giving no dominant cluster but a small fraction of folded structures under equilibrium conditions, possibly due to backbone torsion parameterization and problems in describing local conformational preferences [41, 42]. In particular, folding simulations of this peptide using the 99SB*-ILDN force field showed that the Trpzip-2 *β*-hairpin formation could not be stabilized, as well as that there is an increase in *a*-helix propensity due to an imbalance of the local conformational preferences [41, 42]. The authors state that this discrepancy is caused due to the optimization of the force field using only a single set of backbone torsions for all non-Pro/Gly amino acids, thus precluding the simultaneous fitting of the different intrinsic conformational preferences of various amino acid residues [41, 42]. This suggests the probability of a force field bias towards *α*-helical conformations, and therefore of an inadequate balance between various secondary structure elements, thus making the stabilization of the *β*-hairpin unachievable.

Given the long and successful history of applications of the AMBER99SB family of force fields to peptide folding [25, 69], we decided to perform one additional simulation of only the **hp**_**NG**_**-1** peptide, but this time using the 99SB-ILDN force field instead of the 99SB*-ILDN variant. Results from the aforementioned studies on the Trpzip-2 showed that although 99SB-ILDN underestimates Δ*Η*_F_, it achieves a relatively better folding of the peptide [42]. The aim of this additional simulation was to establish whether the apparent force field deficiency concerning mirror-image *β*-turns was introduced in the force field during its optimization to better sample *α*-helical structures. Analysis of this 99SB-ILDN trajectory with the PROMOTIF program and comparison with the results shown in Table 2 clearly indicated that this is not the case: the cumulative percentages for the I’ and II’ turns remain virtually unchanged between the two force fields. To put this in numbers, the observed changes for the various I’ and II’ turns (compared with the **hp**_**NG**_**-1** entries in Table 2), are: **I’**: 0.41→0.47%, 2.34→2.54%, 0.38→0.31%, **II’**: 0.17→0.08%, 0.04→0.03%, 7.35→6.75%, implying that there is no profound variation in the performance between the star-corrected force field and its uncorrected counterpart. This is not necessarily an unexpected outcome. Despite the success of the 99SB-ILDN variant (which does not account for the helical bias) in folding Trpzip-2 in previous studies, the increased flexibility of our systems leads correspondingly to an increased difficulty of tracking these systems computationally. Note, however, that this remark should not be taken trivially. Constantly increasing computing power has concomitantly facilitated the application of high quality, longer simulations (in the scale of μs, for instance, or even longer). At least for such short peptides, such long simulation times eliminate obscurities associated with insufficient sampling and allow these studies to be seen as an acid test for the ability of force fields to successfully interpret physical reality. In this spirit, the above reported discrepancies add one more piece of evidence to the existing literature by showing that two force fields give a totally different view contrary to QM and X-ray determined results. This, by extent, points towards the necessity of numerous independent case studies for such systematic inaccuracies to be detected, despite the long-term validation a force field might have undergone.

Having noted, though, the above conclusions and aiming to provide the full scenery of the complications accompanying this work, should we solely attribute such discrepancies to the parametrization of the force field? The comparison between DFT and MD rises and falls with the quality of the DFT calculations as well. The differences between these two methods, especially in terms of the potential energy surface calculation, are widely known to the scientific community, and disagreement is not unexpected. Despite the approximations used in MD simulations, the reliability of QM calculations is also not unquestionable. Concerning our case, although the hybrid meta-GGA M06-2X functional [70] coupled with the SMD implicit solvent [71] used by Kang and Yoo for the DFT calculations (in ref 24) have shown a promising performance in determining the structure of very small molecules and peptides [72, 73], additional improvements are necessary especially for efficiently calculating dispersion interactions, which are of utmost importance to the structural stability of biological macromolecules. It has already been demonstrated that the Minnesota functionals do not properly describe medium- and long-rage London-dispersion interactions [74–78]. Even by including empirical dispersion correction parameters (29 fitted parameters for the M06-2X), the calculations’ accuracy is sensitively dependent on the basis set quality. DFT approximations combined with small double-*ζ* basis sets, such as the 6-31G(d) used in ref 24, frequently suffer from inaccuracies due to basis-set superposition (BSSE) and basis-set incompleteness (BSIE) errors. These problems affect not only energies but also properties derived from them such as geometries, as previous studies on peptides and protein fragments have already indicated [76–78]. Despite the fact that the interplay between DFT dispersion corrections and BSSE is a well-known complication, neither of these issues has been addressed by Kang and Yoo in their study. Besides, the use of a more accurate level of theory (namely DSD-PBEP86-D3BJ/def2-TZVP) was implemented for calculating single-point energies rather than geometries. In addition to this, implicit solvation of the PCM type, more precisely the SMD water model, is certainly not adequate to describe specific interactions between a peptide and its hydration shell, which might be of importance for such flexible systems. Explicit microsolvation by a sufficient number of water molecules should at least be considered for such calculations. In the absence of a critical evaluation of their DFT calculations, apart from not allowing for assessing the performance of modern state-of-the-art density functionals, one may cast doubts on the validity of their results and the structures generated. Taking these facts into account, profound deviations between the two methods regarding very specific structural motifs, such as the Asn-Gly *β*II’ turn in our case, may account for inconsistencies in the DFT calculations that were performed. That being the case, we could argue that our MD analysis might after all adequately provide a physically correct description of the structures and dynamics of the Asn-Gly heptapeptides.

## Conclusions

The primary aim of this communication was to evaluate the ability of the AMBER99SB*-ILDN force field to reproduce the structures of turn-forming peptides which were previously characterized by QM-DFT calculations. The DFT calculations indicated that the type I’ *β*-turn is the most preferred motif in aqueous solution for the three heptapeptides containing the Asn-Gly segment. The picture painted by our molecular dynamics analysis, however, is fundamentally different: according to the simulations, the peptides are highly flexible with multiple relatively shallow free energy minima that allow the peptides to quickly interconvert between structurally diverse conformations. The dPCA-based free energy landscapes in Fig 2 and the corresponding cluster analysis show that there is no funnel-like gradient leading to a native state and, thus, there are no prominent and persistent highly populated structures. The apparent higher stability οf the peptides’ central four-residue part does not invalidate the general conclusion about the highly dynamic behavior of these systems. Having noted those differences between QM and MD, we must not fail to also stress their fundamental similarity: these peptides do show a very strong preference for *β*-turn formation. As Table 2 shows, the various types of *β*-turns containing Asn/Gly as one of their central residues occupy the largest part of the respective MD trajectories with cumulative frequencies reaching *≈*85% for two of the peptides (becoming *≈*45% if the type IV turns are excluded). The residue-specific preferences for positions *i*+1 and *i*+2 on the *β*-turns vary significantly between the four central residues, with the Asn-Gly segment preferring the aforementioned positions almost equally with segments formed by neighboring residues (X_-1_-Asn and Gly-X_+1_). Type I *β*-turns appear to be the most prominent MD-derived motif in each trajectory, while Asn-Gly *β*I’ turns show relatively significant populations only for the **hp**_**NG**_**-2** and **hp**_**NG**_**-3** peptides. In agreement with the dPCA results, the torsion angle analysis also demonstrated the structural malleability of the peptides and their pronounced tendency to form various types of *β*-turns. But it was also this more detailed analysis that allowed us to convincingly demonstrate the inability of the AMBER99SB*-ILDN force field to sample the Asn-Gly *β*II’ motif. This apparent force field discrepancy was also confirmed through a comparison between MD and the ideal *β*-turn values obtained by the Thornton group. This comparison (exemplified in Table 4) showed a very reasonable agreement between the two sets with the pronounced exception of the *ψ*Asn angles for the *β*II’ turns. The force field discrepancy has been attributed to its poor parametrization deriving backbone corrections, resulting in the incapability of fully capturing the different intrinsic conformational preferences of various amino acid residues. Hence, one additional simulation was performed with another AMBER99SB variant, based on its previous success in folding the Asn-Gly turn-forming Trpzip-2, giving similar results.

Other than the structural and computational interest on the peptides’ structural preferences and the corresponding comparison between our MD and the available QM-DFT data, the work reported here has clear implications concerning the validation of the force fields, and especially the number of case studies and computational effort required to discover any yet unidentified problems with new force fields. An inescapable conclusion from this work is that just because a force field has successfully been used to study the folding of a large number of peptides, this alone neither implies nor guarantees the absence of systematic errors that would require the application of very specific structural motifs and peptides for these errors to become obvious. Similarly, even for the more rigorous QM calculations, one is encouraged to perform project-specific validation calculations using various density functionals accompanied by different basis sets, to finally select the most appropriate combination, so that robust and meaningful results can be drawn. Having noted this conclusion, we feel that we should also argue against it: our analyses and comparisons were not performed against solid experimental data (obtained, for example, from an NMR study of the peptides), and can thus be argued that we could be completely wrong in assuming that these specific peptides should actually sample, for example, the *β*II’ turn conformation. Having said that, the amount of publications on the Asn-Gly containing peptides, and especially previous NMR studies [17–20, 23, 63] and statistical analyses of protein database [1–5, 7] that have shown the preference of the Asn-Gly sequence for “mirror-image” *β*-turns, leaves little doubt that what we have here is a case where a minor force field bias precludes the formation of the experimentally expected structural motifs. Seeing our results in a negative light, it could be argued that this is a clear reminder that as the pace of producing and publishing new molecular dynamics force fields is increasing, the probability that any of these force fields can be reasonably validated with a multitude of structurally independent case studies is correspondingly decreasing, to the point of making the choice of a suitable force field an exercise in futility.

## Supporting information

S1 Table Structural analysis and *β*-turns occupancy in the dPCA derived clusters. (PDF)

S2 Table Z-scores and corresponding probabilities for the observed deviations between the reference DFT and the MD-derived torsion angles. (PDF)

S1 Fig Extent of sampling and statistical significance. Results from the application of Good-Turing statistics to the three trajectories, for both the full-length peptides and their four-residue central part, obtained using only structures associated with temperatures ≤ 360 K.
(TIF)

S2 Fig *φ*,*ψ angles of* hp_NG_-1 DFT-obtained *β*-turns. Ramachandran plots showing the *φ*,*ψ* torsion angle values of residues *i* to *i*+3 for (A) *β*Ι, (Β) *β*I’, (C) *β*II and (D) *β*II’ DFT-obtained hp_NG_-1 turn structures. Areas in red, yellow, beige and white represent the core, the allowed, the generous and the disallowed regions respectively. Non-glycine residues are depicted here with black square signs and glycine residues with black triangles. Figures were generated using PROCHECK.
(TIF)

S3 Fig *φ*,*ψ angles of* hp_NG_-2 DFT-obtained *β*-turns. Ramachandran plots showing the *φ*,*ψ* torsion angle values of residues *i* to *i*+3 for (A) *β*Ι, (Β) *β*I’, (C) *β*II and (D) *β*II’ DFT-obtained hp_NG_-2 turn structures. Areas in red, yellow, beige and white represent the core, the allowed, the generous and the disallowed regions respectively. Non-glycine residues are depicted here with black square signs and glycine residues with black triangles. Figures were generated using PROCHECK.
(TIF)

S4 Fig *φ*,*ψ angles of* hp_NG_-3 DFT-obtained *β*-turns. Ramachandran plots showing the *φ*,*ψ* torsion angle values of residues *i* to *i*+3 for (A) *β*Ι, (Β) *β*I’, (C) *β*II and (D) *β*II’ DFT-obtained hp_NG_-3 turn structures. Areas in red, yellow, beige and white represent the core, the allowed, the generous and the disallowed regions respectively. Non-glycine residues are depicted here with black square signs and glycine residues with black triangles. Figures were generated using PROCHECK.
(TIF)

S5 Fig *φ*,*ψ torsion angles analysis of* hp_NG_-1 dPCA clusters. Results from the torsion angles analysis of the five most prominent dPCA clusters of hp_NG_-1 for every turn type. Shown from left to right is the number of the cluster along with its population, the occupancy of *β*-turn motifs in each cluster, the histograms showing the distribution of deviations (in degrees) between the reference DFT *φ*,*ψ* values of the 4-residue central part and the respective *φ*,*ψ* values obtained from the simulation, and the corresponding backbone representative structures of each cluster. Representatives with random coil conformation are depicted here only with their Ala-Asn-Gly-Ala part.
(TIFF)

S6 Fig *φ*,*ψ torsion angles analysis of* hp_NG_-2 dPCA clusters. Results from the torsion angles analysis of the five most prominent dPCA clusters of hp_NG_-2 for every turn type. Shown from left to right is the number of the cluster along with its population, the occupancy of *β*-turn motifs in each cluster, the histograms showing the distribution of deviations (in degrees) between the reference DFT *φ*,*ψ* values of the 4-residue central part and the respective *φ*,*ψ* values obtained from the simulation, and the corresponding backbone representative structures of each cluster. Representatives with random coil conformation are depicted here only with their Val-Asn-Gly-Gln part.
(TIFF)

S7 Fig *φ*,*ψ torsion angles analysis of* hp_NG_-3 dPCA clusters. Results from the torsion angles analysis of the five most prominent dPCA clusters of hp_NG_-3 for every turn type. Shown from left to right is the number of the cluster along with its population, the occupancy of *β*-turn motifs in each cluster, the histograms showing the distribution of deviations (in degrees) between the reference DFT *φ*,*ψ* values of the 4-residue central part and the respective *φ*,*ψ* values obtained from the simulation, and the corresponding backbone representative structures of each cluster. Representatives with random coil conformation are depicted here only with their Val-Asn-Gly-Leu part.
(TIFF)

S8 Fig Occupancy of *β*-turns in dPCA clusters. The above barcharts show the occupancy of Asn-Gly *β*-turns among a set of 500 randomly selected structures from every dPCA cluster of the three heptapeptides. Clusters in which *β*-turns were not identified among the set of structures are omitted. Shown at the horizontal axis are clusters’ numbers and the different turn types. The vertical axis shows the % occupancy of the different turn types in each cluster. The structural assignment was performed using the PROMOTIF program.
(TIFF)
