# Supplementary material for: A molecular dynamics simulation study on the propensity of Asn-Gly-containing heptapeptides towards β-turn structures: Comparison with ab initio quantum mechanical calculations
Source: PLoS One. 2020 Dec 3;15(12):e0243429. doi: 10.1371/journal.pone.0243429 (PMC7714341; doi:10.1371/journal.pone.0243429)
Supplement: S1 Table — (PDF) [file pone.0243429.s001.pdf]

S1 Table

S1 Table. Structural analysis and β-turns occupancy in the dPCA derived clusters.

	No. of Cluster	P_i (%)	X_{-1}-Asn	Asn-Gly	Gly-X_{+1}
hp$_{NG}$-1	1	11.88	IV (0.1%)	-	**II' (4.0%)** / IV (1.4%)
	2	4.26	-	**I (2.7%)** / IV (0.1%)	I (1.1%) / IV (1.1%)
	3	8.65	I (2.4%) / IV (1.5%)	**I (3.1%)** / IV (1.1%)	I (1.9%) / VIII (0.1%) / IV (1.2%)
	4	7.23	-	-	II (0.2%) / **IV (0.3%)**
	5	7.67	**I (5.0%)** / IV (2.0%)	IV (0.2%)	I' (0.1%) / IV (0.6%)
	6	6.32	VIII (0.4%) / IV (0.3%)	-	**II' (1.5%)** / IV (0.8%)
	7	5.16	**VIII (0.4%)** / IV (0.3%)	-	II (0.2%) / IV (0.3%)
	8	10.74	-	-	**I (4.6%)** / VIII (0.2%) / IV (2.3%)
	9	9.40	-	**II (6.6%)** / IV (1.5%)	I' (0.1%) / IV (1.7%)
	10	5.10	**I (2.6%)** / IV (1.9%)	IV (0.3%)	II (0.1%) / IV (0.2%)
	11	6.64	**I (3.6%)** / IV (2.1%)	-	II' (1.1%) / IV (0.6%)
	12	5.97	VIII (0.7%) / IV (0.3%)	-	**I (2.5%)** / VIII (0.2%) / IV (1.2%)
	13	2.30	-	**IV (0.2%)**	II (0.1%) / IV (0.1%)
	14	2.75	VIII (0.2%) / IV (0.1%)	**II (1.3%)** / IV (0.6%)	IV (0.5%)
	15	3.01	II (0.1%) / IV (1.0%)	**I' (2.0%)**	IV (0.2%)
	16	2.06	-	-	**II' (0.3%)** / IV (0.2%)
	17	0.67	-	-	**IV (0.1%)**
	18	0.37	**I' (0.1%) / IV (0.1%)**	**I' (0.1%)**	-
hp$_{NG}$-2	1	20.10	II (0.7%) / IV (8.2%)	**I' (9.9%)** / IV (0.3%)	IV (6.8%)
	2	14.09	I (3.8%) / IV (2.6%)	**I (4.3%)** / IV (2.3%)	I (2.7%) / VIII (0.1%) / IV (2.4%)
	3	4.55	-	**I (2.4%)** / IV (0.1%)	I (1.0%) / IV (1.2%)
	4	9.54	**I (6.9%)** / IV (1.9%)	IV (0.4%)	I' (0.1%) / IV (0.7%)
	5	10.02	-	-	**I (5.1%)** / VIII (0.2%) / IV (2.6%)
	6	10.13	VIII (0.2%) / IV (0.2%)	-	**II' (5.8%)** / IV (0.9%)
	7	3.24	**I (1.7%)** / IV (1.3%)	IV (0.3%)	IV (0.2%)
	8	5.98	VIII (0.4%) / IV (0.3%)	-	**I (2.6%)** / VIII (0.2%) / IV (1.4%)
	9	7.55	-	**II (4.6%)** / IV (1.2%)	IV (2.4%)
	10	4.39	VIII (0.1%) / IV (0.2%)	**II (1.8%)** / IV (1.1%)	IV (0.6%)
	11	2.42	**VIII (0.2%) / IV (0.2%)**	-	IV (0.1%)
	12	2.41	-	-	**II (0.1%) / IV (0.1%)**
	13	2.85	**I (1.9%)** / IV (0.7%)	-	II' (0.3%) / IV (0.2%)
	14	2.44	-	-	**II' (0.3%) / IV (0.2%)**
	15	0.28	-	-	-

	1	20.66	I (3.8%) / IV (2.4%)	**I (6.2%)** / IV (1.6%)	I (2.9%) / VIII (0.1%) / IV (3.2%)
	2	17.38	**I (14.0%)** / IV (2.7%)	IV (0.5%)	I' (0.1%) / IV (0.8%)
	3	16.50	II (0.9%) / IV (7.7%)	**I' (9.9%)** / IV (0.1%)	IV (5.2%)
	4	4.22	-	**I (2.3%)**	I (0.9%) / IV (1.1%)
	5	11.33	-	-	**I (6.0%)** / VIII (0.2%) / IV (3.1%)
	6	6.92	-	**II (3.4%)** / IV (1.3%)	IV (1.9%)
	7	7.48	VIII (0.1%) / IV (0.1%)	**II (4.8%)** / IV (1.2%)	IV (2.4%)
**hp$_{NG}$-3**	8	3.87	VIII (0.4%) / IV (0.2%)	-	**I (1.7%)** / VIII (0.1%) / IV (1.0%)
	9	2.65	-	-	**II' (0.3%) / IV (0.3%)**
	10	2.71	-	-	**IV (0.1%)**
	11	1.40	I (1.0%) / **IV (0.3%)**	IV (0.1%)	-
	12	1.74	**I (1.2%)** / IV (0.4%)	-	II' (0.1%) / IV (0.1%)
	13	1.64	**VIII (0.1%) / IV (0.1%)**	-	**IV (0.1%)**
	14	1.08	**VIII (0.1%) / IV (0.1%)**	-	**II' (0.1%) / IV (0.1%)**
	15	0.41	-	-	-

From left to right are listed the number of clusters, its population probability in (%), and the occupancy of different turn types in each cluster in relation to different combinations of i+1 and i+2 turn residues from the peptides' central four-residue part. The assignment of β-turns was performed using the PROMOTIF program. Types VIa1, VIa2 and VIb are excluded from our analysis as they require a Pro residue in position i+2. The most populated turn type in each cluster is highlighted in bold.
