# Supplementary material for: A molecular dynamics simulation study on the propensity of Asn-Gly-containing heptapeptides towards β-turn structures: Comparison with ab initio quantum mechanical calculations
Source: PLoS One. 2020 Dec 3;15(12):e0243429. doi: 10.1371/journal.pone.0243429 (PMC7714341; doi:10.1371/journal.pone.0243429)
Supplement: S2 Table — (PDF) [file pone.0243429.s002.pdf]

S2 Table

S2 Table. Z-scores and corresponding probabilities for the observed deviations between the reference DFT and the MD-derived torsion angles.

	hp$_{NG}$-1			hp$_{NG}$-2			hp$_{NG}$-3		
	**Torsion angle**	**Z-score**	**Probability**	**Torsion angle**	**Z-score**	**Probability**	**Torsion angle**	**Z-score**	**Probability**
***β*I**	***φ*Ala**	-0.63	0.53	***φ*Val**	-1.33	0.18	***φ*Val**	0.06	0.95
	***ψ*Ala**	-0.62	0.54	***ψ*Val**	0.49	0.62	***ψ*Val**	-1.36	0.17
	***φ*Asn**	-1.25	0.21	***φ*Asn**	-1.12	0.26	***φ*Asn**	-1.11	0.27
	***ψ*Asn**	0.60	0.55	***ψ*Asn**	0.52	0.60	***ψ*Asn**	-0.10	0.92
	***φ*Gly**	1.04	0.30	***φ*Gly**	0.31	0.76	***φ*Gly**	2.98	0.00
	***ψ*Gly**	-0.25	0.80	***ψ*Gly**	1.02	0.31	***ψ*Gly**	-1.18	0.24
	***φ*Ala**	1.81	0.70	***φ*Gln**	2.53	0.01	***φ*Leu**	1.89	0.06
	***ψ*Ala**	0.30	0.76	***ψ*Gln**	-0.14	0.89	***ψ*Leu**	0.12	0.90
***β*I'**	***φ*Ala**	0.78	0.44	***φ*Val**	0.29	0.77	***φ*Val**	0.99	0.32
	***ψ*Ala**	3.52	0.00	***ψ*Val**	1.14	0.25	***ψ*Val**	1.20	0.23
	***φ*Asn**	-1.32	0.19	***φ*Asn**	-0.66	0.51	***φ*Asn**	-0.64	0.52
	***ψ*Asn**	-0.99	0.32	***ψ*Asn**	-0.77	0.44	***ψ*Asn**	-1.07	0.28
	***φ*Gly**	-1.84	0.06	***φ*Gly**	-1.42	0.16	***φ*Gly**	-0.29	0.77
	***ψ*Gly**	0.41	0.68	***ψ*Gly**	0.90	0.37	***ψ*Gly**	0.08	0.94
	***φ*Ala**	0.21	0.83	***φ*Gln**	0.22	0.82	***φ*Leu**	-0.66	0.51
	***ψ*Ala**	3.51	0.00	***ψ*Gln**	2.12	0.03	***ψ*Leu**	-2.26	0.02
***β*II**	***φ*Ala**	-0.76	0.45	***φ*Val**	-1.17	0.24	***φ*Val**	0.04	0.97
	***ψ*Ala**	-0.53	0.60	***ψ*Val**	-0.38	0.70	***ψ*Val**	-1.29	0.20
	***φ*Asn**	-1.50	0.13	***φ*Asn**	-1.53	0.13	***φ*Asn**	-1.62	0.10
	***ψ*Asn**	0.55	0.58	***ψ*Asn**	0.83	0.41	***ψ*Asn**	0.45	0.65
	***φ*Gly**	-0.39	0.70	***φ*Gly**	-1.33	0.18	***φ*Gly**	0.76	0.45
	***ψ*Gly**	0.06	0.95	***ψ*Gly**	0.68	0.50	***ψ*Gly**	-1.07	0.28
	***φ*Ala**	1.57	0.12	***φ*Gln**	2.03	0.04	***φ*Leu**	1.97	0.05
	***ψ*Ala**	1.40	0.16	***ψ*Gln**	1.09	0.28	***ψ*Leu**	0.86	0.39
***β*II'**	***φ*Ala**	0.67	0.50	***φ*Val**	0.14	0.89	***φ*Val**	0.40	0.69
	***ψ*Ala**	3.31	0.00	***ψ*Val**	1.18	0.24	***ψ*Val**	3.13	0.00
	***φ*Asn**	-1.41	0.16	***φ*Asn**	-0.58	0.56	***φ*Asn**	-0.16	0.87
	***ψ*Asn**	-4.06	0.00	***ψ*Asn**	-4.02	0.00	***ψ*Asn**	-3.46	0.00
	***φ*Gly**	-0.04	0.97	***φ*Gly**	1.24	0.21	***φ*Gly**	-0.37	0.71
	***ψ*Gly**	0.16	0.87	***ψ*Gly**	0.23	0.82	***ψ*Gly**	0.90	0.37
	***φ*Ala**	0.39	0.70	***φ*Gln**	-0.46	0.64	***φ*Leu**	-1.31	0.19
	***ψ*Ala**	3.64	0.00	***ψ*Gln**	2.14	0.03	***ψ*Leu**	-0.14	0.89
