# Supplementary figures and images for: A molecular dynamics simulation study on the propensity of Asn-Gly-containing heptapeptides towards β-turn structures: Comparison with ab initio quantum mechanical calculations
Source: PLoS One. 2020 Dec 3;15(12):e0243429. doi: 10.1371/journal.pone.0243429 (PMC7714341; doi:10.1371/journal.pone.0243429)

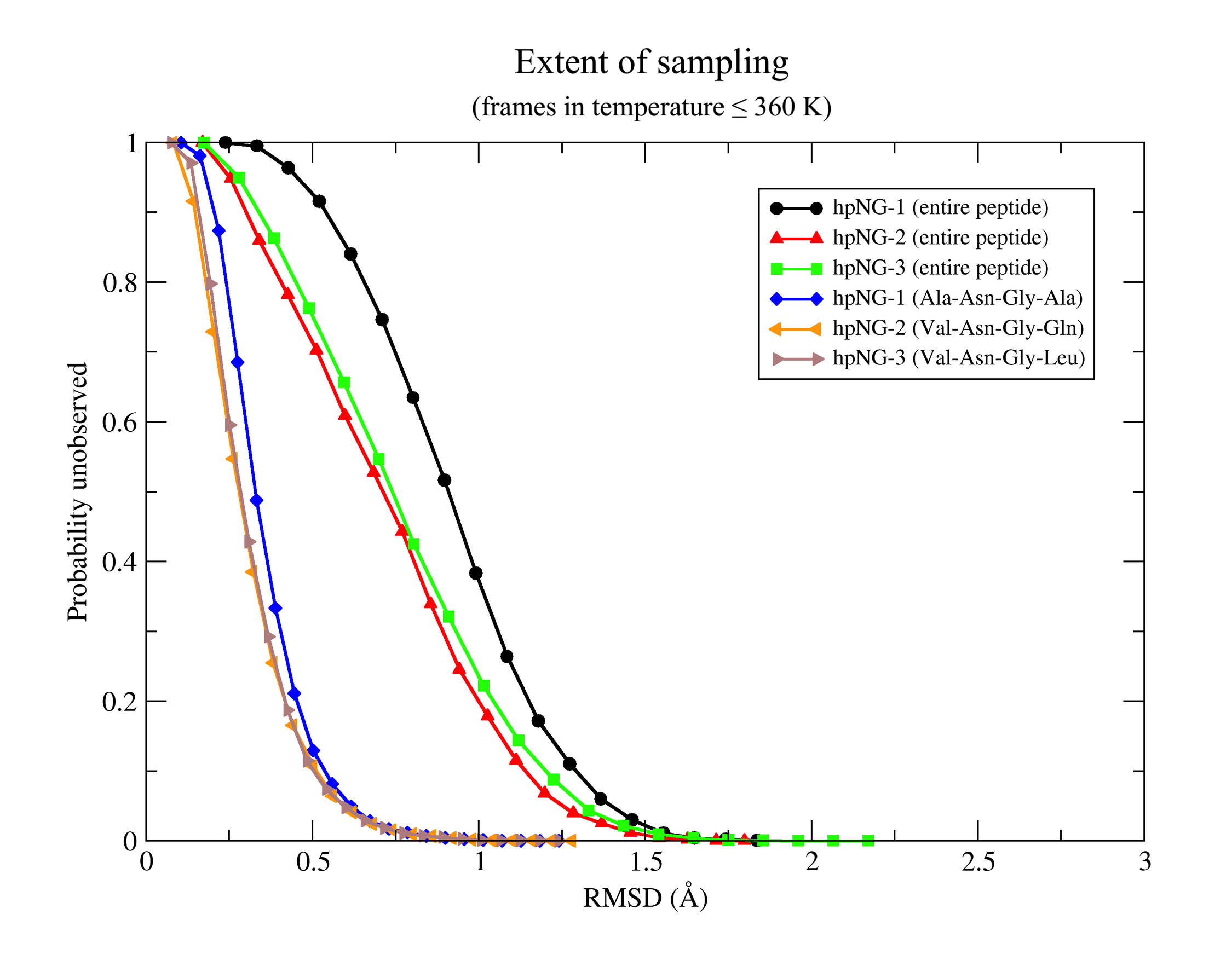

Supplement: S1 Fig — Results from the application of Good-Turing statistics to the three trajectories, for both the full-length peptides and their four-residue central part, obtained using only structures associated with temperatures ≤ 360 K. (TIF) [file pone.0243429.s003.tif]

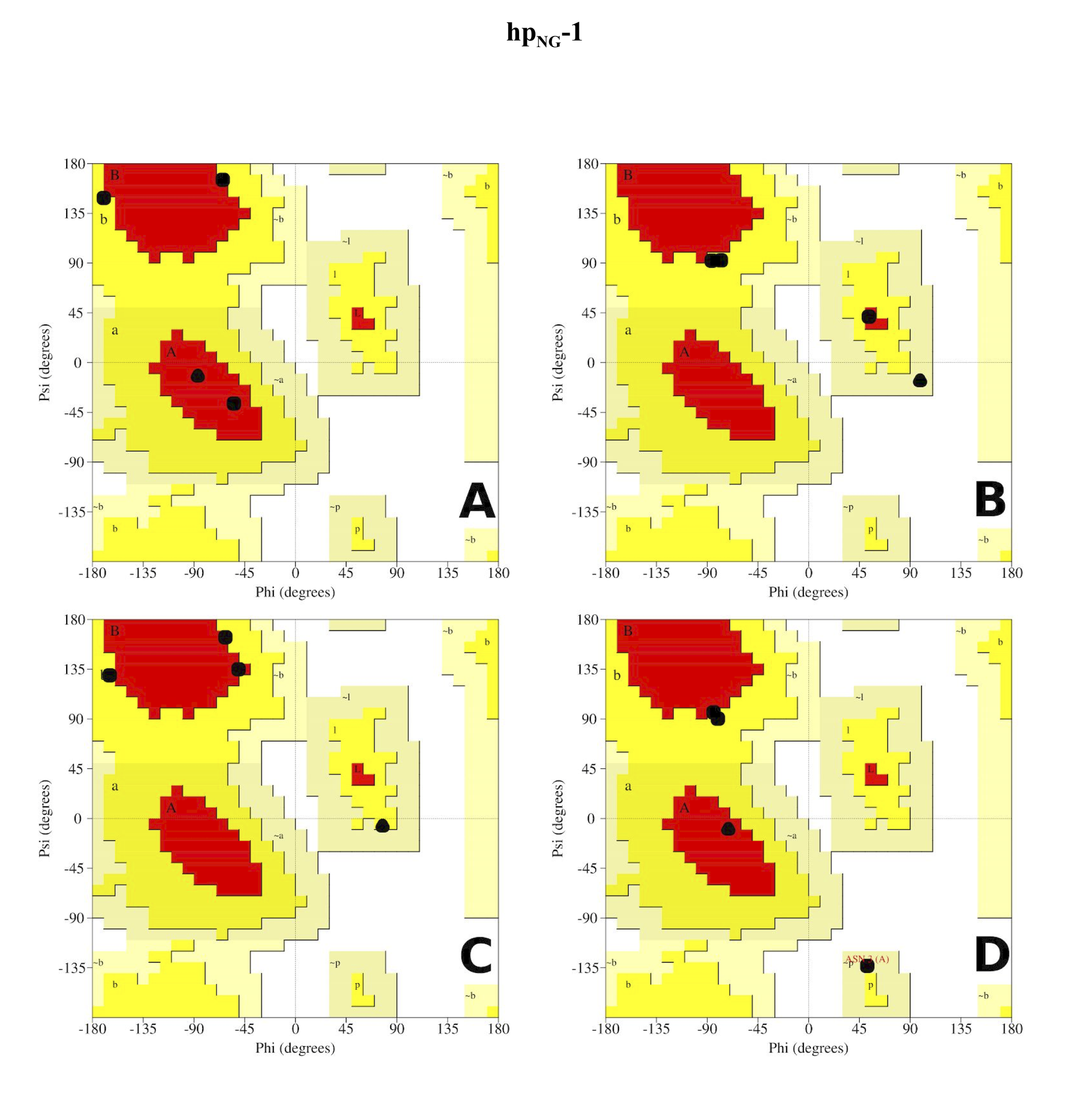

Supplement: S2 Fig — Ramachandran plots showing the φ,ψ torsion angle values of residues i to i+3 for (A) βΙ, (Β) βI’, (C) βII and (D) βII’ DFT-obtained hpNG-1 turn structures. Areas in red, yellow, beige and white represent the core, the allowed, the generous and the disallowed regions respectively. Non-glycine residues are depicted here with black square signs and glycine residues with black triangles. Figures were generated using PROCHECK. (TIF) [file pone.0243429.s004.tif]

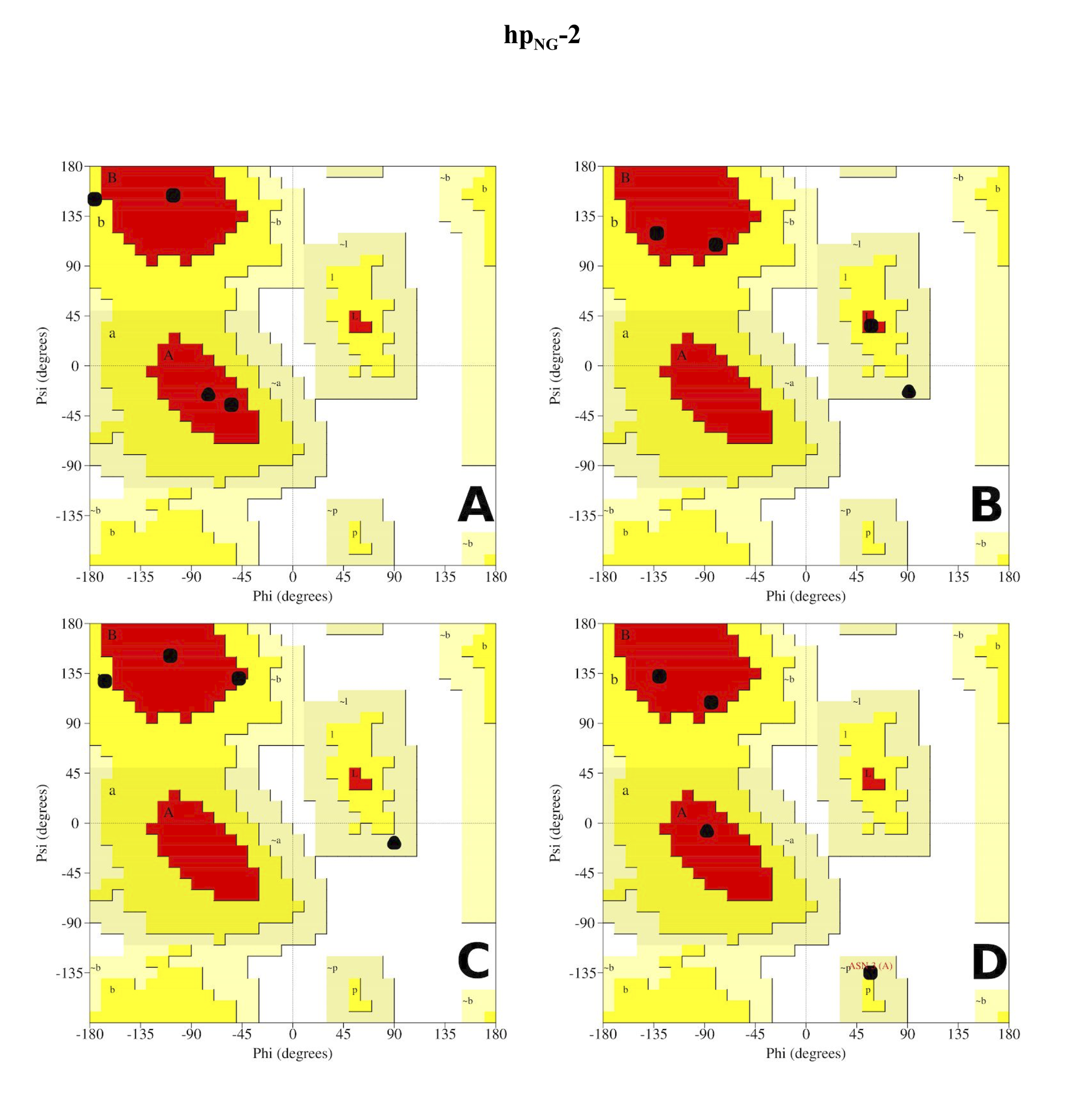

Supplement: S3 Fig — Ramachandran plots showing the φ,ψ torsion angle values of residues i to i+3 for (A) βΙ, (Β) βI’, (C) βII and (D) βII’ DFT-obtained hpNG-2 turn structures. Areas in red, yellow, beige and white represent the core, the allowed, the generous and the disallowed regions respectively. Non-glycine residues are depicted here with black square signs and glycine residues with black triangles. Figures were generated using PROCHECK. (TIF) [file pone.0243429.s005.tif]

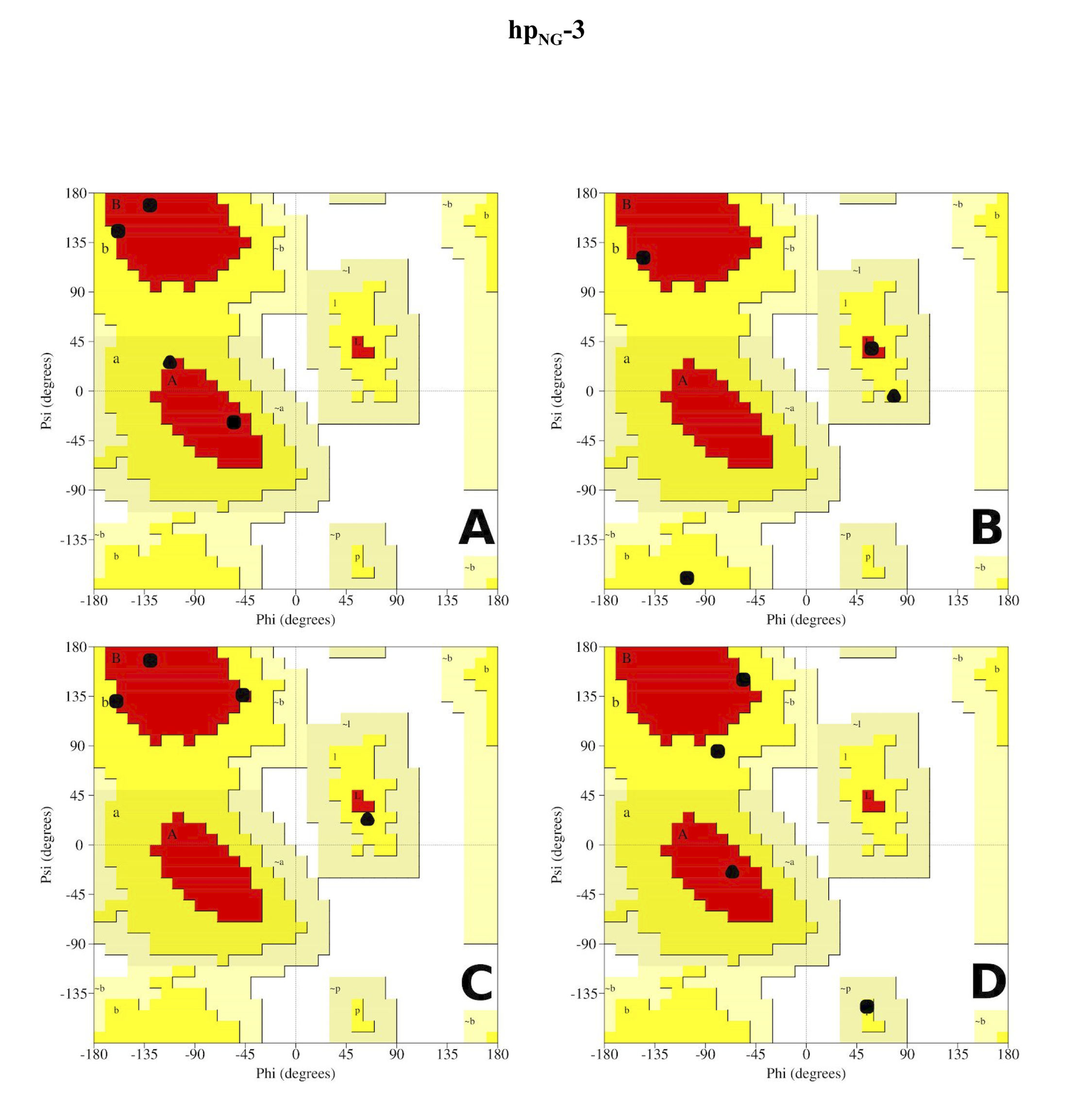

Supplement: S4 Fig — Ramachandran plots showing the φ,ψ torsion angle values of residues i to i+3 for (A) βΙ, (Β) βI’, (C) βII and (D) βII’ DFT-obtained hpNG-3 turn structures. Areas in red, yellow, beige and white represent the core, the allowed, the generous and the disallowed regions respectively. Non-glycine residues are depicted here with black square signs and glycine residues with black triangles. Figures were generated using PROCHECK. (TIF) [file pone.0243429.s006.tif]

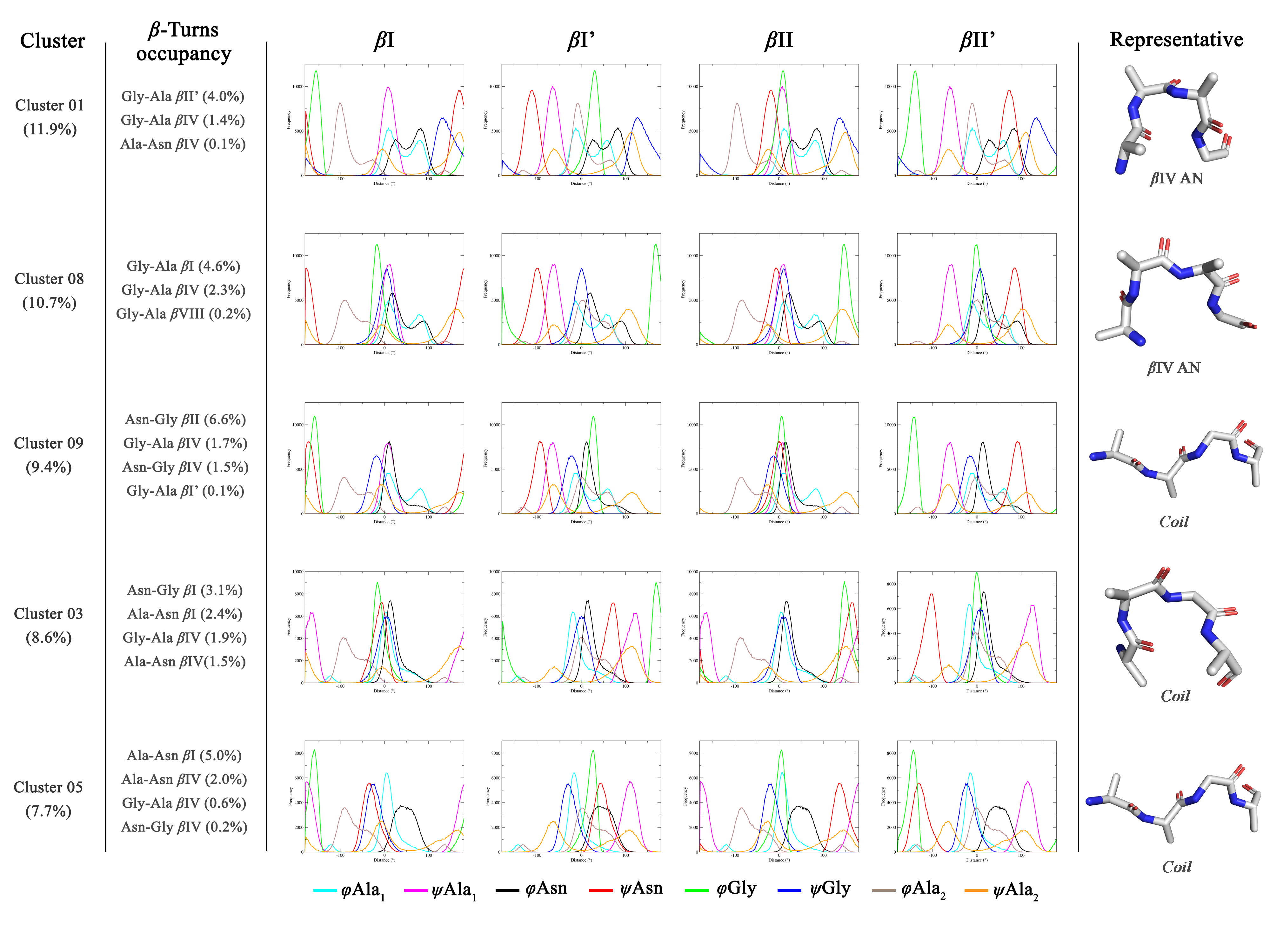

Supplement: S5 Fig — Results from the torsion angles analysis of the five most prominent dPCA clusters of hpNG-1 for every turn type. Shown from left to right is the number of the cluster along with its population, the occupancy of β-turn motifs in each cluster, the histograms showing the distribution of deviations (in degrees) between the reference DFT φ,ψ values of the 4-residue central part and the respective φ,ψ values obtained from the simulation, and the corresponding backbone representative structures of each cluster. Representatives with random coil conformation are depicted here only with their Ala-Asn-Gly-Ala part. (TIFF) [file pone.0243429.s007.tiff]

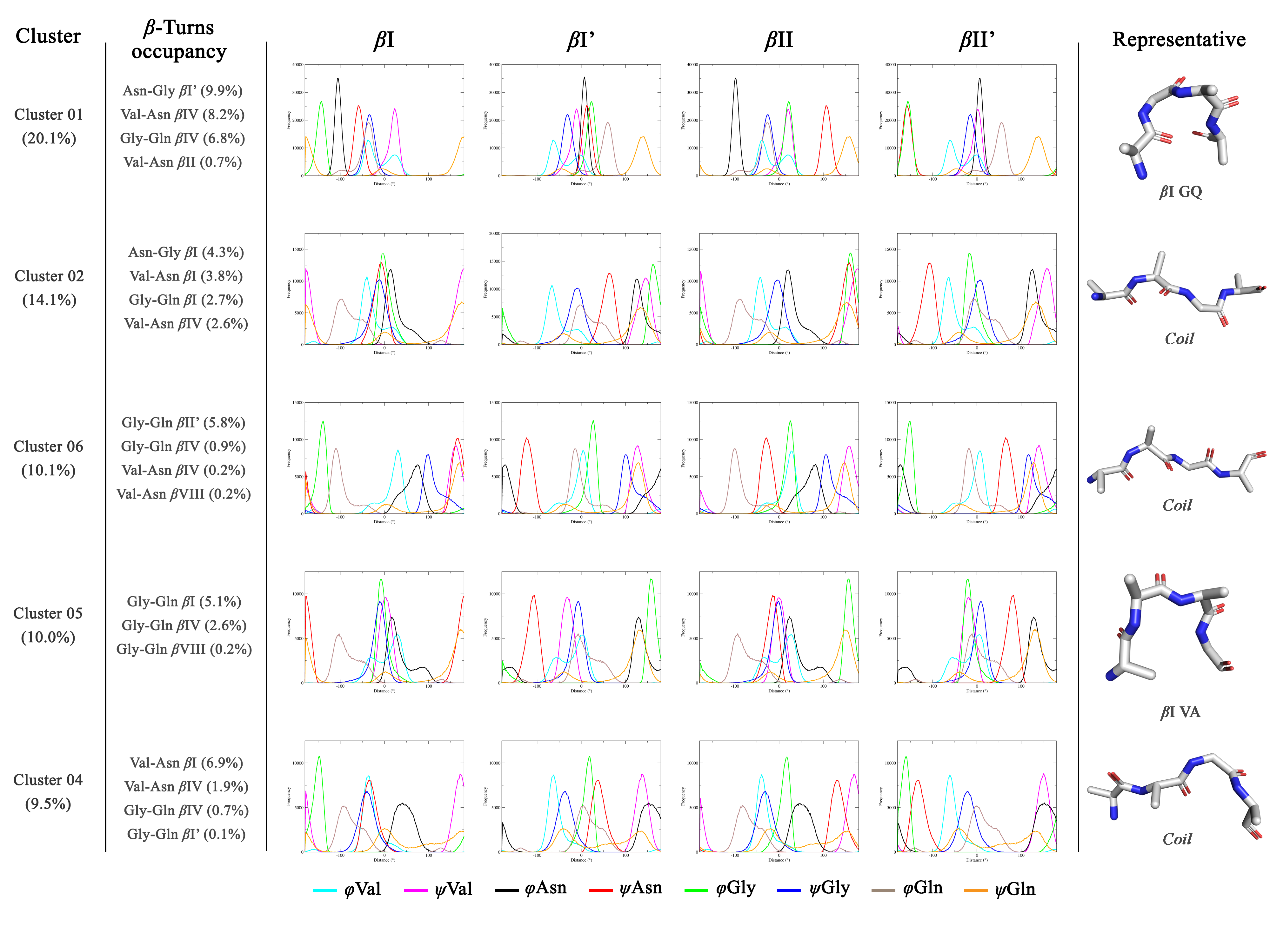

Supplement: S6 Fig — Results from the torsion angles analysis of the five most prominent dPCA clusters of hpNG-2 for every turn type. Shown from left to right is the number of the cluster along with its population, the occupancy of β-turn motifs in each cluster, the histograms showing the distribution of deviations (in degrees) between the reference DFT φ,ψ values of the 4-residue central part and the respective φ,ψ values obtained from the simulation, and the corresponding backbone representative structures of each cluster. Representatives with random coil conformation are depicted here only with their Val-Asn-Gly-Gln part. (TIFF) [file pone.0243429.s008.tiff]

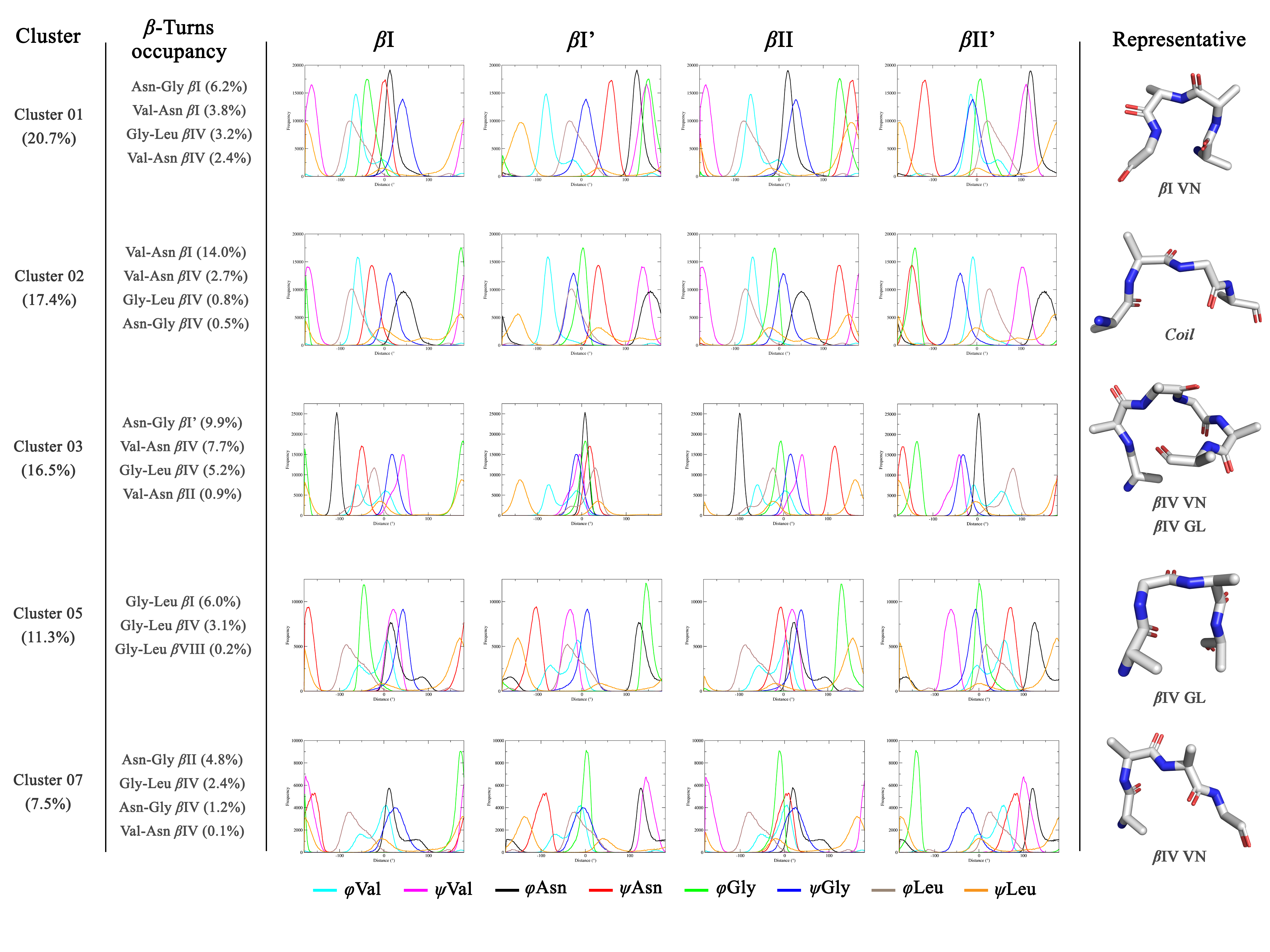

Supplement: S7 Fig — Results from the torsion angles analysis of the five most prominent dPCA clusters of hpNG-3 for every turn type. Shown from left to right is the number of the cluster along with its population, the occupancy of β-turn motifs in each cluster, the histograms showing the distribution of deviations (in degrees) between the reference DFT φ,ψ values of the 4-residue central part and the respective φ,ψ values obtained from the simulation, and the corresponding backbone representative structures of each cluster. Representatives with random coil conformation are depicted here only with their Val-Asn-Gly-Leu part. (TIFF) [file pone.0243429.s009.tiff]

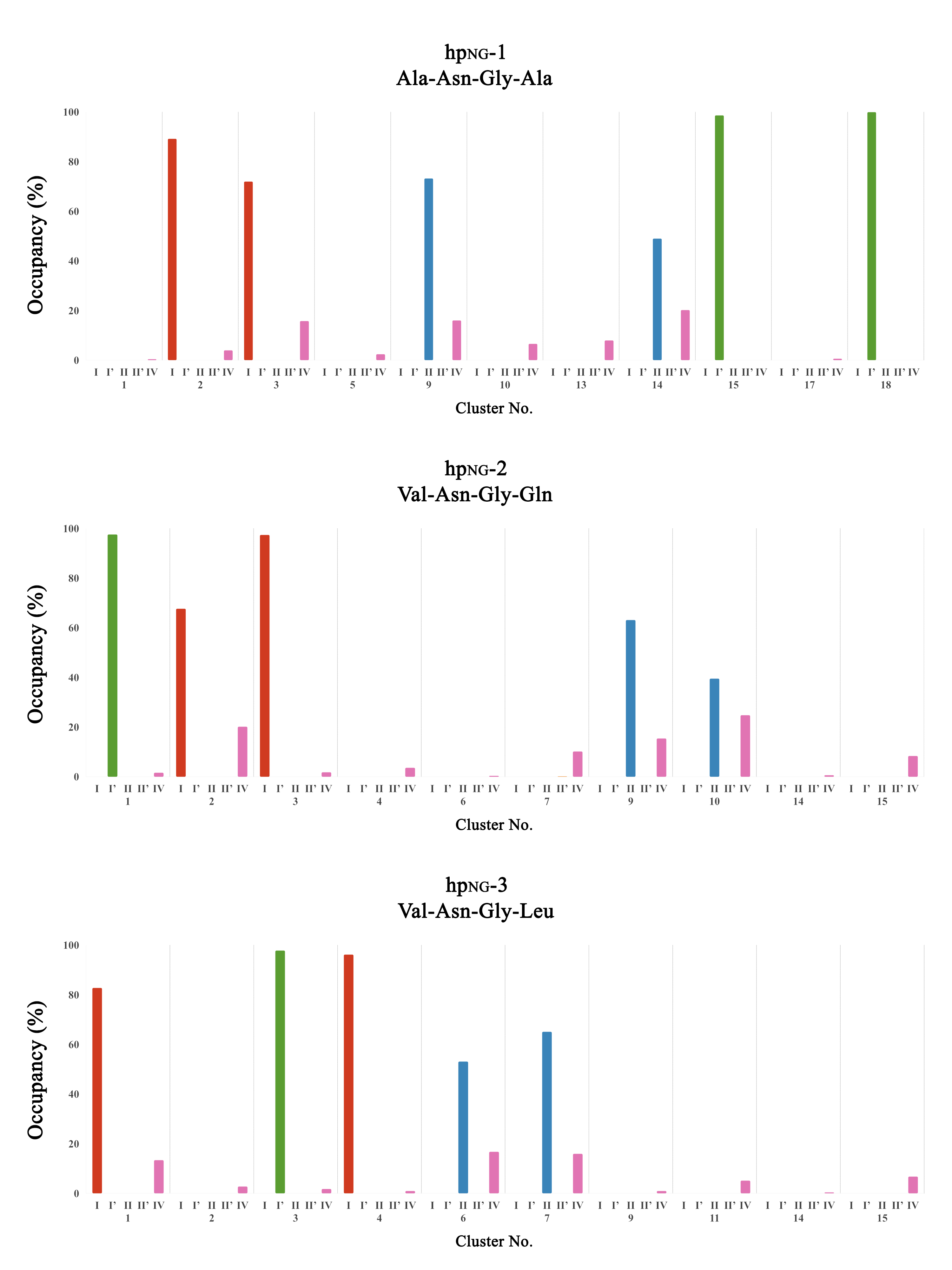

Supplement: S8 Fig — The above barcharts show the occupancy of Asn-Gly β-turns among a set of 500 randomly selected structures from every dPCA cluster of the three heptapeptides. Clusters in which β-turns were not identified among the set of structures are omitted. Shown at the horizontal axis are clusters’ numbers and the different turn types. The vertical axis shows the % occupancy of the different turn types in each cluster. The structural assignment was performed using the PROMOTIF program. (TIFF) [file pone.0243429.s010.tiff]
